# Influence of Terminal Functionality on the Crystal Packing Behaviour and Cytotoxicity of Aromatic Oligoamides

**DOI:** 10.3389/fchem.2021.709161

**Published:** 2021-06-30

**Authors:** Pierre Delfosse, Colin C. Seaton, Louise Male, Rianne M. Lord, Sarah J. Pike

**Affiliations:** ^1^ School of Chemistry and Biosciences, University of Bradford, Bradford, United Kingdom; ^2^ School of Chemistry, University of Birmingham, Birmingham, United Kingdom; ^3^ School of Chemistry, University of East Anglia, Norwich Research Park, Norwich, United Kingdom

**Keywords:** aromatic oligoamides, cytotoxicity, crystallography, terminal group, breast and ovarian cancer

## Abstract

The synthesis and characterization of three aromatic oligoamides, constructed from the same pyridyl carboxamide core but incorporating distinct end groups of acetyl (Ac) **1**, *tert*-butyloxycarbonyl (Boc) **2** and amine **3** is reported. Single crystal X-ray diffraction analysis of **1**–**3** and a dimethylsulfoxide (DMSO) solvate of **2** (**2**-DMSO), has identified the presence of a range of intra- and intermolecular interactions including N-H⋯N, N-H⋯O=C and N-H⋯O=S(CH_3_)_2_ hydrogen-bonding interactions, C-H⋯π interactions and off-set, face-to-face stacking π-π interactions that support the variety of slipped stack, herringbone and cofacial crystal packing arrangements observed in **1**–**3**. Additionally, the cytotoxicity of this series of aromatic oligoamides was assessed against two human ovarian (A2780 and A2780cisR), two human breast (MCF-7 and MDA-MB-231) cancerous cell lines and one non-malignant human epithelial cell line (PNT-2), to investigate the influence of the terminal functionality of these aromatic oligoamides on their biological activity. The chemosensitivity results highlight that modification of the terminal group from Ac to Boc in **1** and **2** leads to a 3-fold increase in antiproliferative activity against the cisplatin-sensitive ovarian carcinoma cell line, A2780. The presence of the amine termini in **3** gave the only member of the series to display activity against the cisplatin-resistance ovarian carcinoma cell line, A2780cisR. Compound **2** is the lead candidate of this series, displaying high selectivity towards A2780 cancer cells when compared to non-malignant PNT-2 cells, with a selectivity index value >4.2. Importantly, this compound is more selective towards A2780 (*cf.* PNT-2) than the clinical platinum drugs oxaliplatin by > 2.6-fold and carboplatin by > 1.6-fold.

## Introduction

The rise of cancer cell resistance towards clinical anticancer drugs, combined with the poor selectivity they can demonstrate for cancers over non-malignant tissue and the occurrence of adverse side-effects, has driven the search for new compounds with increased antiproliferative activity and selectivity. (Mader et al., 1998; Winocur et al., 2006; Figaro et al., 2011; Tageja et al., 2011; Ward et al., 2021). Whilst there is a diverse array of anticancer agents currently used in the clinic, small organic molecules, for example, lenalidoamide and flutamide, represent an important group of chemotherapeutic agents. Aromatic oligoamides (Hamuro et al., 1994; Hamuro et al., 1996; Yuan et al., 2004; Yuan et al., 2005; Kortelainen et al., 2015) are a class of small organic compounds that have been shown to possess potential anticancer activity (Tew et al., 2002; Ernst et al., 2003; Yin and Hamilton, 2005; Davis et al., 2007; Plante et al., 2009; Azzarito et al., 2012; Burslem et al., 2014; Jayatunga et al., 2014; Burslem et al., 2016) and have also been employed in a wide range of applications including catalysis, (Hegedus et al., 2019), sensing, (Yi et al., 2005; Bao et al., 2008; Yamato et al., 2009), materials chemistry (König et al., 2000; Garía et al., 2010) and crystal engineering. (Suhonen et al., 2016; Annala et al., 2017).

Systematic solid-state studies of aromatic oligoamides have identified that small structural variations in these molecules can have a profound influence on their conformational behavior and such studies can to help deepen our understanding of their structure-activity relationships (SARs). A crystallographic study of aromatic oligoamides by Nissinen and co-workers (Suhonen et al., 2012) showed that modification of the aromatic ring from benzene to pyridine results in marked changes in the folding behavior of these compounds resulting in the adoption of curved molecular structures. Gunnlaugsson and co-workers described a crystallographic analysis of a series of cytotoxic pyridine-based aromatic oligoamides, showing that they adopted curved molecular structures with a supramolecular arrangement that could potentially promote interaction with DNA. (Frimannsson et al., 2010). The pyridine-based aromatic oligoamides were identified as DNA-targeting supramolecular binders and displayed cytotoxicity against the drug-resistant chronic myeloid leukaemia, K562 cell line. Fletcher and co-workers determined SARs on a series of short chain aromatic oligoamides, highlighting that relaxation of the rigidity of the backbone of the scaffold lead to increased cytotoxicity. (Yap et al., 2012). The lead candidate of the series displays low IC_50_ values (1.1–4.3 μM) against the human colon carcinoma (DLD-1), mesothelioma (I45), lung carcinoma (A549), and human non-small cell lung carcinoma (H1299).

**FIGURE 1:**
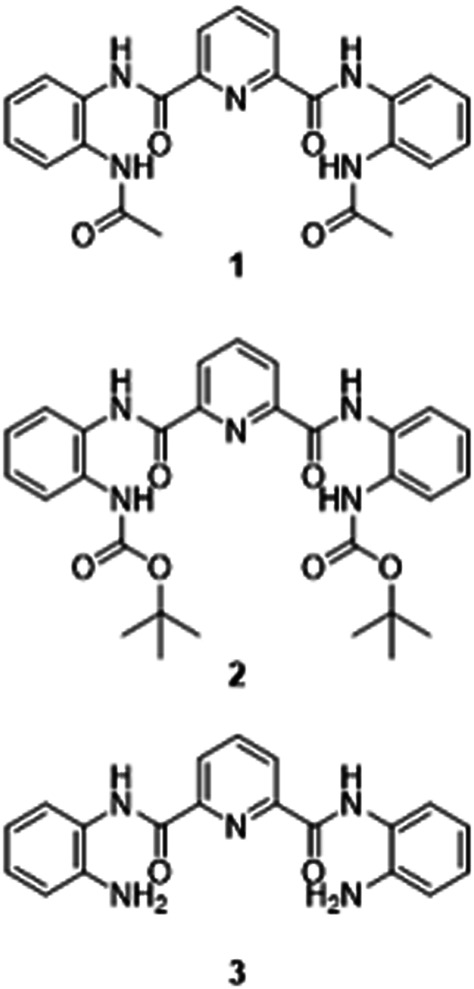
Short chain aromatic oligoamides **1–3** employed in this study.

Gaining an understanding of the influence of the structure of an aromatic oligoamide on its biological activity is central to the development of new molecules within this class that have the potential to demonstrate improved cytotoxicity towards cancerous cells. To probe the influence of the terminal group on the solid-state structure and antiproliferative activity of these aromatic oligoamides, we undertook the synthesis, crystallographic analysis and cytotoxicity studies of three aromatic oligoamides based on the same pyridyl carboxamide core but including different end groups; acetyl (Ac) **1**, *tert*-butyloxycarbonyl (Boc) **2** and amine **3** (Figure 1). We report on the solid-state properties of **1**–**3** and solvatomorph **2**-DMSO, and employ single-crystal X-ray diffraction analysis to identify the presence of a range of non-covalent interactions which support the diverse crystal packing behavior of these aromatic oligoamides. We describe the influence of varying the terminal functionality in compounds **1**-**3** on their cytotoxicity against breast and ovarian cancer cell lines, and report the chemosensitivity studies against a non-malignant cell type. The results show that the most promising compound, a Boc-terminated aromatic oligoamide, is non-toxic towards non-malignant cells, unlike all cisplatin (**CDDP**), carboplatin (**CARB**) and oxaplatin (**OXA**), which all demonstrate high cytotoxicity.

## Results and Discussion

Aromatic oligoamides, **1**–**3**, which all have the same pyridyl carboxamide core, but incorporate different terminal groups of Ac **1**, Boc **2** and NH_2_ **3** (Figure 1) have been prepared according to known or modified literature procedures, (Annala et al., 2017; Suhonen et al., 2016; Suhonen et al., 2012) (Suhonen et al., 2012; Suhonen et al., 2016; Annala et al., 2017), and were all characterized by ^1^H and ^13^C{^1^H} NMR spectroscopy, melting point analysis, FTIR spectroscopy, high-resolution mass spectrometry and single crystal X-ray diffraction. The ^1^H and ^13^C NMR spectra of **1**–**3** indicate that these compounds are symmetrical, with the ^1^H NMR spectra showing only one resonance for the NHs in the amide bonds of the terminal Ac and Boc group of **1** and **2** at δ 10.93 and δ 10.73 ppm respectively. Whilst the ^13^C NMR spectrum of **3** displays only one resonance for the 2 C atoms in the carbonyl groups adjacent to the pyridine ring at δ 161.2 ppm (see Supporting Information). Electronspray ionization mass spectrometry identified the molecular ion peaks at *m*/*z* 432.1670 [M + H]^+^ **1**, 548.2503 [M + H]^+^ **2** and 348.1453 [M + H]^+^ **3**.

### Crystallographic Studies

Single crystals suitable for X-ray diffraction were obtained for **1**–**3** and for a DMSO solvatomorph of **2** (**2**-DMSO). Table 1 summarizes selected crystallographic data for **1**–**3** and **2**-DMSO (for full crystallographic tables, see Supporting Information). X-ray diffraction analysis identified the nature of the non-covalent interactions present in the solid state for each of the studied aromatic oligoamides.

**TABLE 1:** Selected crystallographic data for **1, 2, 2**-DMSO and **3**.

	1	2	2-DMSO	3
Empirical formula	C_23_H_21_N_5_O_4_	C_29_H_33_N_5_O_6_	C_31_H_39_N_5_O_7_S	C_38_H_34_N_10_O_4_
Formula weight	431.45	547.60	625.73	694.75
Crystal system	Monoclinic	Orthorhombic	Monoclinic	Monoclinic
Space group	*P*2_1_/c	*P*2_1_2_1_2_1_	*P*2_1_/c	*P*2_1_/c
*a*/Å	4.8617 (2)	9.9736 (7)	9.3413 (3)	16.114 (15)
*b*/Å	18.2381 (7)	14.9397 (11)	17.6116 (7)	13.297 (12)
*c*/Å	22.8681 (6)	19.4229 (15)	19.7290 (7)	17.625 (16)
*α*/°	90	90	90	90
*β*/°	93.870 (3)	90	96.048 (2)	116.80 (2)
*γ*/°	90	90	90	90
Volume/Å^3^	2023.05 (13)	2894.1 (4)	3227.7 (2)	3371 (5)
Z	4	4	4	4
Temperature/K	100.01	169.99	170.0	170.39
ρ_calc_ g/cm^3^	1.417	1.257	1.288	1.369
μ/mm^−1^	0.823	0.089	0.153	0.093
F (000)	904.0	1160.0	1328.0	1456.0
Radiation	Cu Kα (*λ* = 1.54184)	MoKα (*λ* = 0.71073)	MoKα (*λ* = 0.71073)	MoKα (*λ* = 0.71073)
2Θ range for data collection/°	7.75–145.704	4.91–56.9	4.754–66.276	2.832–55.33
Index ranges	−5 ≤ h ≤ 5, −22 ≤ k ≤ 15, −28 ≤ l ≤ 27	−13 ≤ h ≤ 13, −19 ≤ k ≤ 19, −26 ≤ l ≤ 25	−12 ≤ h ≤ 14, −27 ≤ k ≤ 26, −30 ≤ l ≤ 30	−19 ≤ h ≤ 20, −15 ≤ k ≤ 17, −22 ≤ l ≤ 22
Reflections collected	7,870	65,497	75,624	27,712
Independent reflections	3887 [R_int_ = 0.0202, R_sigma_ = 0.0267]	7,098 [R_int_ = 0.1280, R_sigma_ = 0.1249]	12,237 [R_int_ = 0.0829, R_sigma_ = 0.0712]	7,712 [R_int_ = 0.1203, R_sigma_ = 0.1340]
Data/restraints/parameters	3887/0/307	7,098/0/447	12,237/0/553	7,712/0/578
Goodness-of-fit on F	1.044	1.031	0.999	0.969
Final R indexes [I>=2σ (I)]	R_1_ = 0.0368, wR_2_ = 0.0887	R_1_ = 0.0596, wR_2_ = 0.1058	R_1_ = 0.0523, wR_2_ = 0.1016	R_1_ = 0.0947, wR_2_ = 0.2264
Final R indexes [all data]	R_1_ = 0.0446, wR_2_ = 0.0932	R_1_ = 0.1453, wR_2_ = 0.1295	R_1_ = 0.1143, wR_2_ = 0.1221	R_1_ = 0.2001, wR_2_ = 0.3055
Largest diff. Peak/hole/e Å^−3^	0.22/−0.20	0.23/−0.27	0.36/−0.48	0.34/−0.39

#### Crystallographic Analysis of 1

**FIGURE 2:**
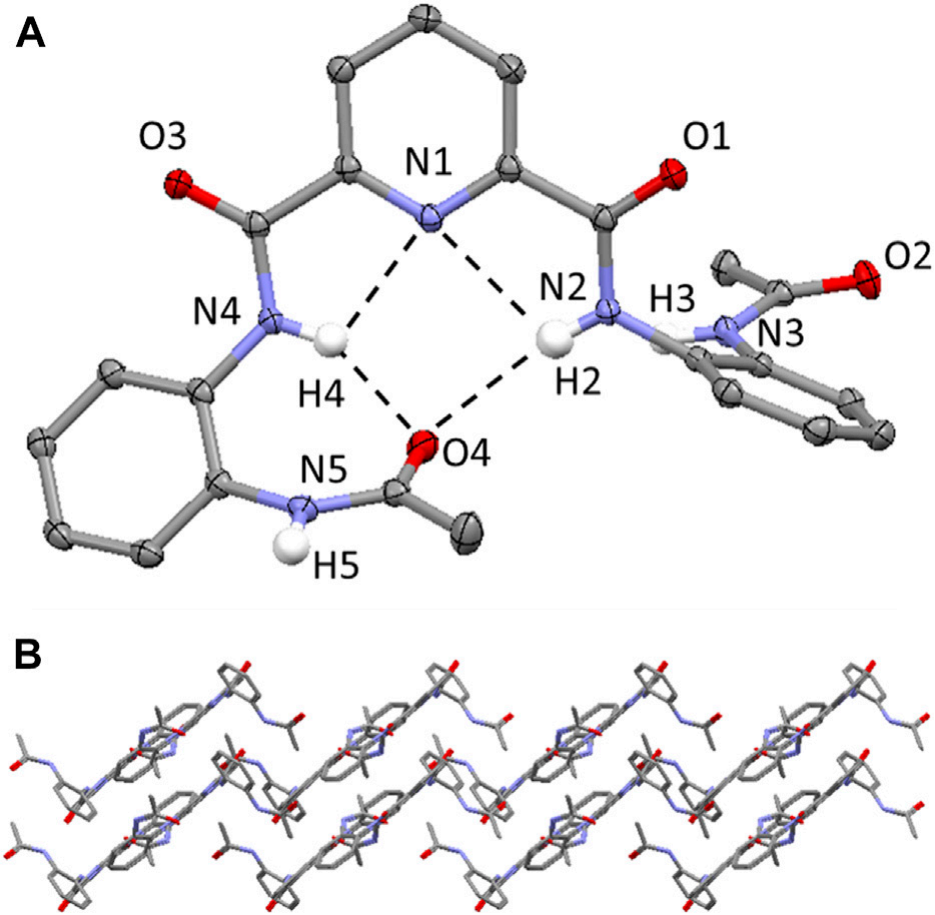
**(A)** Molecular structure of compound **1**. H atoms (expect those on the N atoms of the amide bonds) have been omitted for clarity and displacement ellipsoids are at 50% probability level. The hydrogen bonding interactions are shown as dashed black lines. H atoms are shown in white, C atoms in grey, N in light blue, O in red. **(B)** Crystal packing of **1** highlighting the slipped stacked layered arrangement aligned along the b axis. Hydrogen atoms have been omitted for clarity.

Single crystals of **1** were grown by vapor diffusion of diethyl ether into a dimethylformamide solution at ambient temperature. **1** crystallizes in a monoclinic crystal system and solution refinement was performed in the *P*2_1_/c space group (Table 1). The molecular structure of **1** is shown in Figure 2A, with displacement ellipsoids placed at 50% probability level. **1** displays three sets of bifurcated intramolecular hydrogen-bonding interactions, firstly, involving the pyridyl N atom and the two NH’s of the adjacent amide group (i.e., N (2/4)-H (2/4)⋯N (1) (2.6583(17)-3.2310(16) Å, Table 2) and, additionally, two bifurcated interactions exist between each of the NH’s of a central amide group and the adjacent pyridyl N atom and the O atom of the terminal amide group (N (2/4)-H (2/4A)⋯N (1) and N (2/4)-H (2/4A)⋯O (4) (1.97(2)-2.39(2) Å, Figure 2A). (Rozas et al., 1998) **1** displays a slipped stack crystal packing arrangement, (Yao et al., 2018), aligned along the *b* axis (Figure 2B), which is supported by two sets of intermolecular hydrogen-bonding interactions and one set of edge-to-face π-π stacking interactions. One of the intermolecular hydrogen-bonding interactions is present between one of the NH’s of an terminal Ac group and an O atom on the carbonyl of the central amide group (N (3)-H (3A)⋯O (1) (1.99 (2) Å) and results in the formation of a hydrogen-bond chain orientated along the *c* axis (Supplementary Figure S7). The second intermolecular hydrogen-bonding interaction is present between one of the NH’s of an terminal amide group and the O atom of the carbonyl group of the Ac capping group in an adjacent molecule (N (5)-H (5A)⋯O (2) (2.00 (2) Å, Supplementary Figure S8). **1** also displays an edge-to-face π-π stacking interaction between the terminal 2-acylaminophenyl rings on neighboring molecules, further supporting the slipped stack crystal packing arrangement (Supplementary Figure S9). (Nishio, 2011).

**TABLE 2:** Cytotoxicity values (IC_50_/μm±SD) for cisplatin (CDDP), oxaliplatin (OXA), carboplatin (CARB) and compounds **1–3** after a 96 h incubation period with human ovarian carcinomas (A2780, A2780cisR), human breast adenocarcinomas (MCF‐7, MDA‐MB‐231) and non-malignant prostate cells (PNT-2).^a^ Selective Index (SI) values when compared to PNT-2 are shown in parenthesis.

Compounds	IC_50_ values (μM) ± SD				
	A2780	A2780cisR	MCF-7	MDA-MB-231	PNT-2
CDDP	1.3 ± 0.1 (6.4)	14 ± 1 (0.6)	1.5 ± 0.2 (5.6)	3.07 ± 0.02 (2.8)	8.5 ± 0.4
CARB	17 ± 1 (1.6)	>100 (0.3*)	>100 (0.3*)	33 ± 2 (0.8)	27 ± 2
OXA	0.505 ± 0.002 (2.6)	2.09 ± 0.03 (0.6)	2.6 ± 0.2 (0.5)	2.5 ± 0.6 (0.5)	1.3 ± 0.2
1	77 ± 5 (1.3*)	>100 (nd)	>100 (nd)	63 ± 4 (1.6*)	>100
2	24.0 ± 0.9 (4.2*)	>100 (nd)	84 ± 3 (1.2*)	69 ± 3 (1.4*)	>100
3	>100 (nd)	61 ± 2 (1.6*)	>100 (nd)	>100 (nd)	>100

a All values are averages from duplicate technical repeats and triplicate experimental repeats. * indicates the minimum SI value as at least one IC_50_ value is >100 μM. n. d. (not determined) indicates the values where both IC_50_ values are >100 μM.

#### Crystallographic Analysis of 2 and 2-DMSO

Single crystals of compound **2** and the dimethylsulfoxide (DMSO) solvate, **2-**DMSO, were grown from two different crystallization conditions at ambient temperature, firstly, through the slow evaporation of chloroform to give **2** and secondly, through the slow evaporation of a 9:1 chloroform:DMSO solvent mixture to generate **2**-DMSO. In the former conditions, **2** crystallizes in an orthorhombic crystal system and solution refinement was performed in the *P*2_1_2_1_2_1_ space group (Table 1) and in the latter conditions, **2** crystallizes, as the DMSO solvate, in a monoclinic crystal system and solution refinement was performed in the *P*2_1_/c space group (Table 1).

**FIGURE 3:**
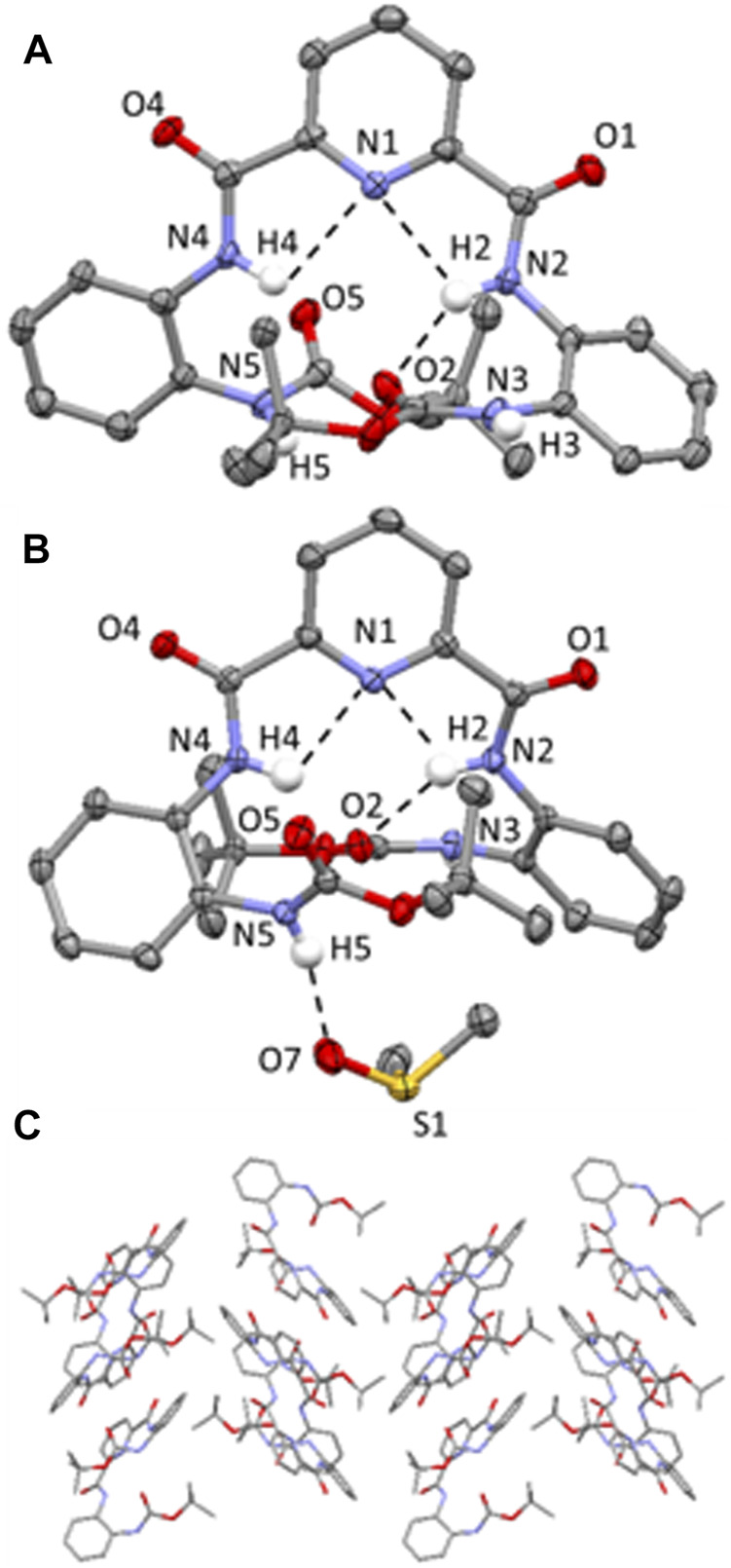
**(A)** Molecular structure of compound **2**; **(B)** Molecular structure of compound **2**-DMSO. H atoms (expect those on the N atoms of the amide bonds) have been omitted for clarity and displacement ellipsoids are at 50% probability level. H atoms are shown in white, C in grey, N in light blue and C in red. Hydrogen-bonding interactions are shown as dashed black lines; **(C)** Crystal packing of **2** highlighting the herringbone stacking arrangement aligned along the b axis. Hydrogen atoms have been omitted for clarity.

The molecular structures of **2** and the **2-**DMSO solvate are shown in Figure 3, with displacement ellipsoids placed at 50% probability level. Both **2** and **2**-DMSO display two sets of bifurcated intramolecular hydrogen bonding interactions, firstly, between the pyridyl N atom and the two NH’s of the adjacent amide groups (N (2/4)-H (2/4A)⋯N (1) 2.34(4)-2.36(4) Å, Figures 3A,B) and, secondly, between one of the NH’s in a central amide group and the adjacent pyridyl N atom and the O atom of the terminal amide group (N (2)-H (2A)⋯N (1) and N (2)-H (2)⋯O (2) (1.88(4)-2.36(4) Å, Figures 3A,B). (Arifuzzaman et al., 2013).

**2** adopts a herringbone crystal packing arrangement (Dhar et al., 2014) aligned along the *b* axis, shown in Figure 3C, and is supported by a range of different intermolecular non-covalent interactions including hydrogen-bonding interactions, edge-to-face π-π stacking interactions and C-H (aryl)⋯π interactions. Two distinct intermolecular N-H⋯O=C hydrogen-bonding interactions are observed in **2**, both of which are orientated along the *a* axis and involve the NH protons of the terminal Boc groups and the carbonyl O atoms on the pyridyl moiety of an adjacent molecule (i.e. N (3)-H (3)⋯O (4) = C and N (5)-H (5)⋯O (1) = C hydrogen bonding interactions, (2.03(4)-2.231(4) Å, Supplementary Figure S12). Additionally, there is an edge-to-face π-π stacking interaction present between the terminal 2-*tert*-butylcarboxyaminophenyl rings on neighboring molecules (Supplementary Figure S13) and a C-H (aryl)⋯π interaction involving an H atom of the Boc group and a terminal 2-*tert*-butylcarboxyaminophenyl ring of an adjacent molecule (Supplementary Figure S14). (Tárkányi et al., 2008).

In the crystal packing of **2**-DMSO, there are two different types of intermolecular hydrogen-bonding interactions present; firstly, there is a N-H⋯O=C interaction between one of the NHs of a terminal Boc group and the O atom on the carbonyl group of a pyridyl amide group (N (3)-H (3)⋯O (4) = C, (2.012 (18) Å, Supplementary Figure S17) and, secondly, there is an intermolecular N-H⋯O=S(CH_3_)_2_ hydrogen-bonding interaction present which involves one of the NH’s of a terminal group moiety and the O atom of a DMSO solvent molecule (N (5)-H (5)⋯O (7) = S(CH_3_)_2_, (2.007 (19) Å, Figure 3B). (Arifuzzaman et al., 2013).

#### Crystallographic Analysis of 3

**FIGURE 4:**
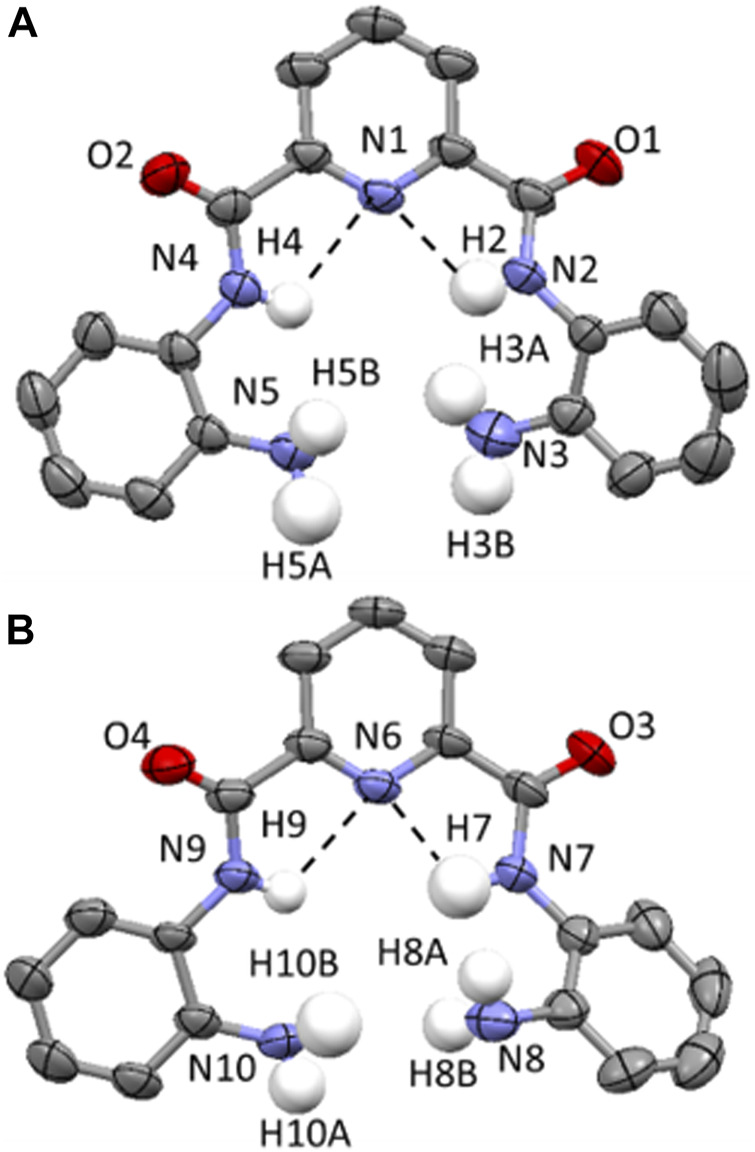
**(A and B)** Molecular structure of two distinct molecules of compound **3** found in the unit cell. Displacement ellipsoids are at 50% probability level. H atoms are shown in white, C in grey, N in light blue and C in red. All H atoms (except for those on the N atoms of the amide and amine functionalities) have been removed for clarity. Hydrogen-bonding interactions are shown as dashed black lines.

Single crystals of compound **3** were grown through the slow evaporation of chloroform at ambient temperature. **3** crystallizes in a monoclinic space group and solution refinement was performed in the *P*2_1_/c space group (Table 1). In the unit cell of **3**, there are two distinct molecules present and the molecular structure is shown in Figure 4 with displacement ellipsoids placed at 50% probability level. Both molecules show the presence of a bifurcated intramolecular hydrogen-bonding interactions involving the pyridyl N atom and the adjacent amide NHs (N (2/4)-H (2/4)⋯N (1) and N (7/9)-H (7/9)⋯N (6), 2.08(5)-2.36 (4 Å, Figure 4). (Arifuzzaman et al., 2013).

**FIGURE 5:**
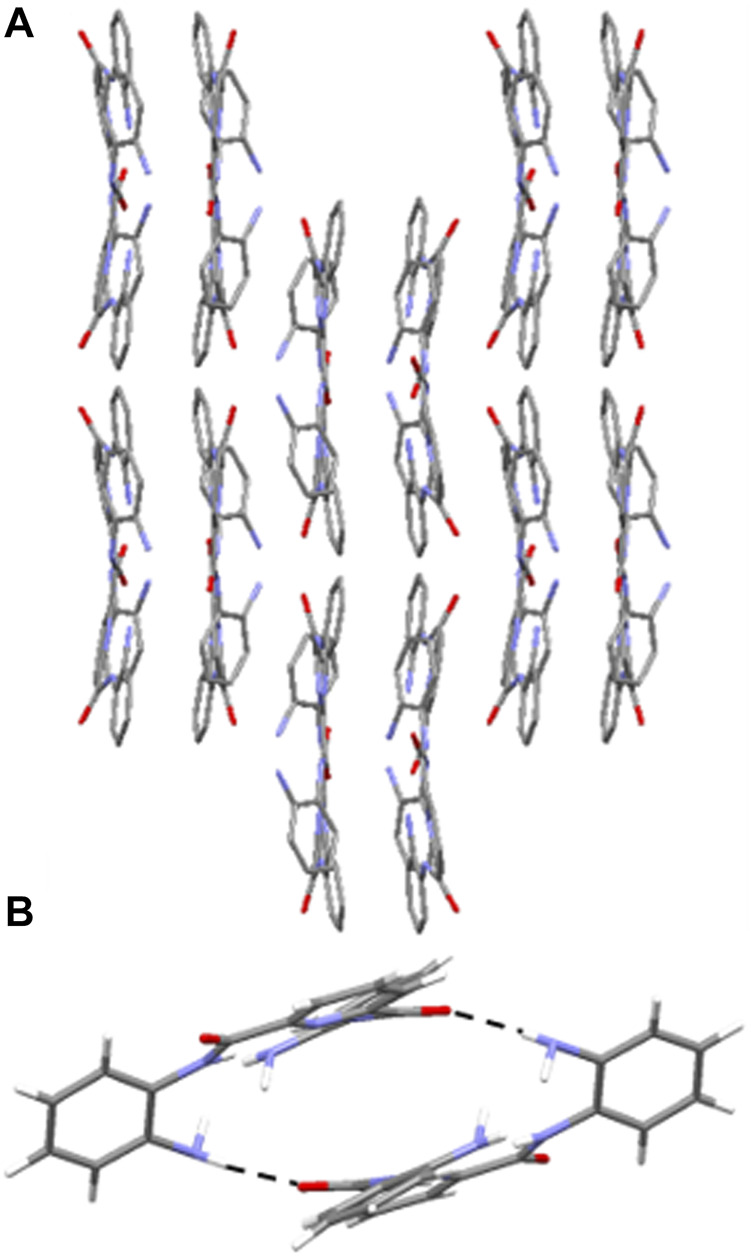
**(A)** Crystal packing of **3** as viewed along the c axis, highlighting the cofacial and slipped layered arrangement. Hydrogen atoms have been omitted for clarity. **(B)** Hydrogen-bonded dimer of **3** observed in the solid state through reciprocal intermolecular N-H⋯O=C hydrogen bonding interactions from the terminal amine NH and the amide O of an adjacent molecule. H atoms are shown in white, C in grey, N in light blue and O in red. Hydrogen-bonding interactions are shown as dashed black lines.

**3** adopts a combination of cofacial and slipped stack layered crystal packing arrangement (Chang et al., 2008; Kobayashi et al., 2006) orientated along the *c* axis (Figure 5A) and this is supported by a series of intermolecular hydrogen-bonding interactions and parallel displaced π-π stacking interactions. In **3**, there are three distinct sets of N-H⋯O=C intermolecular interactions including those observed between the NH of a terminal amine moiety in one molecule and the O atom of the carbonyl group in the amide group of an adjacent molecule (N (3)-H (3B)⋯O (2) 2.15 (7) Å (Supplementary Figure S21), N (8)-H (8A)⋯O (4) 2.06 (5) Å, (Supplementary Figure S22) and N (10)-H (10A)⋯O (3) 2.28 (4) Å (Supplementary Figure S23). The second of which adopts reciprocal intermolecular hydrogen-bonding interactions between two adjacent molecules, giving rise to the formation of a hydrogen-bonded dimer (Figure 5B). Additionally, there are two sets of intermolecular parallel displaced π-π stacking interactions present which support the cofacial and slipped stacking crystal packing arrangement of **3** (Supplementary Figure S24, 25). (Egli et al., 2003).

### Chemosensitivity Studies

**FIGURE 6:**
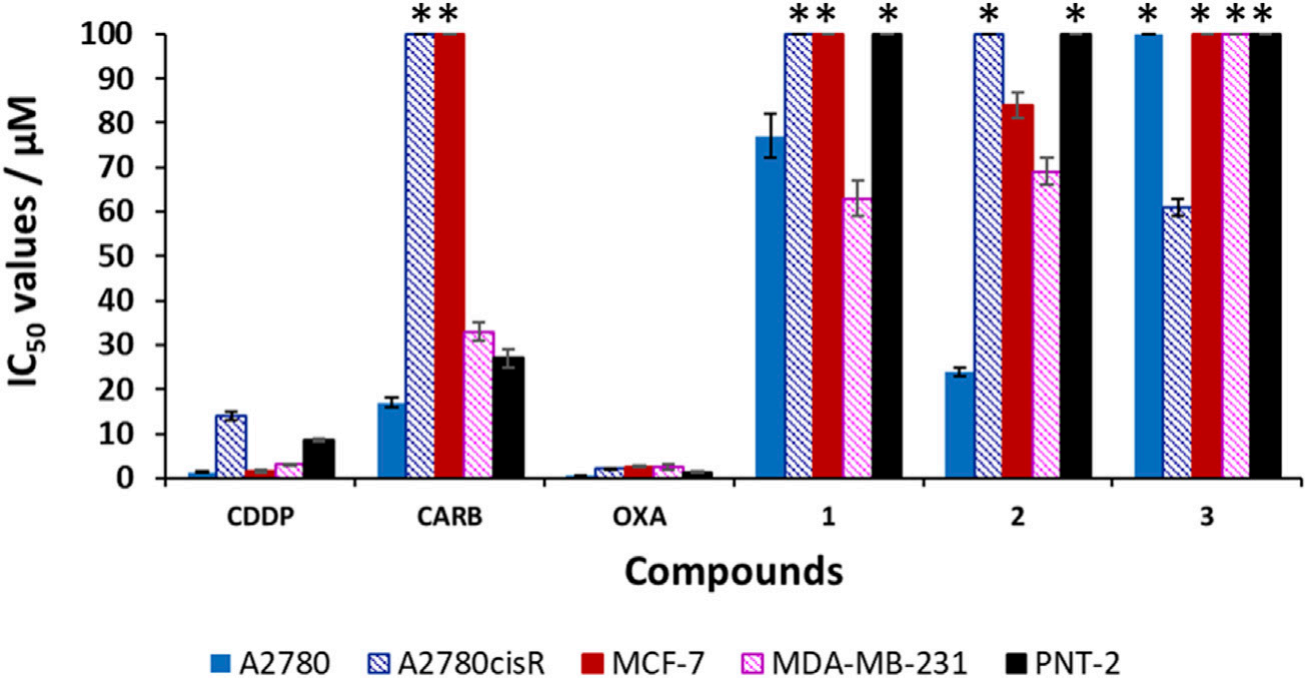
Cytotoxicity values (IC_50_/μM ± SD) for cisplatin (**CDDP**), carboplatin (**CARB**), oxaliplatin (**OXA**) and compounds **1–3** against human cell lines: ovarian carcinomas (A2780, A2780cisR), breast adenocarcinomas (MCF-7, MDA-MB-231) and non-malignant prostate (PNT-2). * indicates that the IC_50_ value is greater than the tested threshold concentration of 100 µM.

Cisplatin (**CDDP**), carboplatin (**CARB**) and oxaliplatin (**OXA**) and compounds **1**–**3** were screened for their cytotoxicity against human cell lines: cisplatin-sensitive ovarian carcinoma (A2780), cisplatin-resistant ovarian carcinoma (A2780cisR) and breast adenocarcinomas (MCF-7 and MDA-MB-231). The IC_50_ values were obtained via the MTT assay after a 96 h incubation period of each compound with the cells at 37°C and 5% CO_2_ (Table 2; Figure 6). The Ac-terminated compound **1** was found be moderate to non-cytotoxic against all cell lines, with IC_50_ values ranging from 63 ± 4 μM to >100 μM. Similarly, the Boc-terminated analogue **2** was found to be moderate to non-cytotoxic against A2780cisR, MCF-7 and MDA-MB-231. However, a significant increase in cytotoxicity is observed when comparing compounds **1** with **2** against A2780, with the potency of **2** increasing by up to 3-fold (77 ± 5 μM for **1** *cf.* 24 ± 0.9 μM for **2**). The amine-terminated compound **3** in non-toxic towards the breast adenocarcinomas cell lines (MCF-7 and MDA-MB-231), with IC_50_ values greater than the tested threshold (>100 μM). Notably, **3** is non-toxic against the cisplatin-sensitive ovarian carcinoma A2780 but is the only one in the library which displays any level of antiproliferative activity against the cisplatin-resistant ovarian carcinoma cell line, A2780cisR, with a moderate IC_50_ value of 61 ± 1 μM.

On analysis of these results no definite structure-activity relationship can be established but a general observation that the nature of the terminal group on these short aromatic oligoamides has a marked effect on determining their cytotoxicity against ovarian carcinomas (A2780 and A2780cisR) and breast adenocarcinomas (MCF-7 and MDA-MB-231). Results highlight the Boc-terminated compound **2** displays the highest activity, with moderate sensitivity against A2780 and the amine-terminated compound **3**, is the only compound to display any level of cytotoxicity against A2780cisR.

### Selectivity Index

**CDDP**, **OXA,** and **CARB** and compounds **1**–**3** were also screened against the non-malignant prostate cell line (immortalized with SV40), PNT-2, to determine any cancer cell selectivity. The results for **CDDP**, **OXA,** and **CARB** show that these clinical platinum drugs have high to moderately cytotoxicity towards PNT-2, with IC_50_ values of 1.3 ± 0.2 μM (**OXA**), 8.5 ± 0.4 μM (**CDDP**) and 27 ± 2 μM (**CARB**). Unlike the clinical platinum drugs, compounds **1–3** are non-toxic towards PNT-2 (IC_50_ values >100 μM) The selectivity index (SI) values were calculated for all the compounds, using the IC_50_ values obtained against PNT-2 and dividing by the IC_50_ value against the cancer cell line in parenthesis in Table 2). A SI value >1 indicates increased selectivity for the cancerous cell line over the non-malignant one, whilst a SI value <1 indicates the inverse (i.e., increased selectivity for the non-malignant cell line over the cancerous one). Compound **1** shows only slight increases in selectivity, with an SI > 1.3* (*p* < 0.05, where * indicates the minimum SI value due to the PNT-2 IC_50_ value >100 μM, Table 2 footnote) for A2780.^1^ However, an SI > 1.6* (*p* < 0.05) for this compound against the triple negative breast cancer (TNBC) cell line, MDA-MB-231, is higher than those observed for the clinical platinum anticancer drugs **OXA** and **CARB** (of 0.5 and 0.8 respectively).^1^ Compound **2** displays a notable SI > 4.2* (*p* < 0.05) against A2780 and a very moderate increase in selectivity towards MCF-7 (SI > 1.2*, *p* < 0.05) and MDA-MB-231 (SI > 1.4*).^1^ The amine-terminated compound **3**, is the only compound to display increased selectivity for A2780cisR when compared to PNT-2, with a SI > 1.6* (*p* < 0.05), which, albeit is very modest, is higher than the SI values observed for **CDDP** (0.6), **OXA** (0.6) and **CARB** (0.3*). Overall, these results highlight that small structural changes to the terminal groups of these aromatic oligoamides can have a marked effect on their biological activity against the tested ovarian and breast cancer cell lines. Herein, it is shown that modification of the terminal groups from Ac to Boc leads to a notable increase in the SI against A2780 but similar SI values are observed against MDA-MD-231, whilst variation of the terminal group to NH_2_ leads to a change in the SI of the aromatic oligoamide against A2780cisR with a slight increase in SI (>1.6*, *p* < 0.05) being observed by this amine-terminated compound.

## Conclusion

In conclusion, we have synthesized and characterized a series of aromatic oligoamides based on a common pyridyl carboxamide core but incorporating distinct end groups: acetyl (Ac) **1**, *tert*-butyloxycarbonyl (Boc) **2** and amine **3**. Single crystal X-ray diffraction analysis of **1**–**3** and **2**-DMSO has identified the presence of an array of non-covalent interactions including N-H⋯N and N-H⋯O=C hydrogen-bonding interactions, a series of C-H⋯π and π-π stacking interactions that support the diverse crystal packing arrangements present in these aromatic oligoamides including slipped stack (**1**), herringbone (**2**) and cofacial/slipped stacked (**3**). The crystal packing of **3** also reveals the presence of hydrogen-bonded dimer formed by the presence of reciprocal intermolecular N-H⋯O=C hydrogen bonding interactions formed between the NH of the terminal amine groups and the O atom on the carbonyl group in the amide group of an adjacent molecule.

To understand SARs, the cytotoxicity of the compound **1**–**3** (and **CDDP**, **OXA** and **CARB**) were obtained via a 96 h MTT assay, and screening against human ovarian carcinomas (A2780 and A2780cisR), human breast adenocarcinomas (MCF-7 and MDA-MB-231) and non-malignant prostrate cell line (PNT-2). Generally, compounds **1**–**3** display either moderate cytotoxicity or are non-toxic against A2780cisR, MCF-7 and MDA-MB-231 cancer cell lines. The Boc-terminated compound, **2**, is the lead candidate of the tested aromatic oligoamides displaying an IC_50_ value of 24 ± 0.9 μM against A2780. Unlike the tested clinical platinum anticancer drugs, compound **2** is non-toxic towards PNT-2 (IC_50_ > 100 µM), meaning it displays an SI value >4.2*-fold towards A2780 (*cf.* PNT-2), making it more selective towards ovarian cancer than the platinum drugs **CDDP** and **OXA** (SI values against A2780: 1.6 (**CDDP**), 6.5 (**CARB**); 2.6 (**OXA**)). The insights gained from this study, regarding the importance of small structural modifications on influencing the biological activity of aromatic oligoamides, will facilitate the future design of related compounds with improved cytotoxicity against ovarian and breast cancer cell lines.

## Data Availability

The datasets presented in this study can be found in online repositories. The names of the repository/repositories and accession number(s) can be found in the article/Supplementary Material.

## Footnotes

1 The IC_50_ values of **1-3** against PNT-2 are greater than the tested threshold concentration of 100 μM and the reported results are minimum SI values (*) and could be greater than reported here.
