# Supplementary material for: Influence of Terminal Functionality on the Crystal Packing Behaviour and Cytotoxicity of Aromatic Oligoamides
Source: Front Chem. 2021 Jun 30;9:709161. doi: 10.3389/fchem.2021.709161 (PMC8277928; doi:10.3389/fchem.2021.709161)

checkCIF/PLATON report

Structure factors have been supplied for datablock(s) 3

THIS REPORT IS FOR GUIDANCE ONLY. IF USED AS PART OF A REVIEW PROCEDURE FOR PUBLICATION, IT SHOULD NOT REPLACE THE EXPERTISE OF AN EXPERIENCED CRYSTALLOGRAPHIC REFEREE.

No syntax errors found. CIF dictionary Interpreting this report

Datablock: 3

```
Bond precision:  C-C = 0.0072 A          Wavelength=0.71073

Cell:            a=16.114(15)     b=13.297(12)       c=17.625(16)
                 alpha=90         beta=116.80(2)     gamma=90
Temperature:     170 K

                Calculated                 Reported
Volume          3371(5)                    3371(5)
Space group     P 21/c                     P 1 21/c 1
Hall group      -P 2ybc                    -P 2ybc
Moiety formula  C19 H17 N5 O2              2(C19 H17 N5 O2)
Sum formula     C19 H17 N5 O2              C38 H34 N10 O4
Mr              347.38                     694.75
Dx,g cm-3       1.369                      1.369
Z               8                          4
Mu (mm-1)       0.093                      0.093
F000            1456.0                     1456.0
F000’           1456.56
h,k,lmax        21,17,23                   20,17,22
Nref            7872                       7712
Tmin,Tmax       0.946,0.970                0.340,0.746
Tmin’           0.943

Correction method= # Reported T Limits: Tmin=0.340 Tmax=0.746
AbsCorr = MULTI-SCAN

Data completeness= 0.980          Theta(max)= 27.665

R(reflections)= 0.0947( 3181)     wR2(reflections)= 0.3055( 7712)

S = 0.969                  Npar= 578
```

The following ALERTS were generated. Each ALERT has the format
test-name_ALERT_alert-type_alert-level.
Click on the hyperlinks for more details of the test.

Alert level C

```
RINTA01_ALERT_3_C  The value of Rint is greater than 0.12
            Rint given   0.120
PLAT026_ALERT_3_C Ratio Observed / Unique Reflections (too) Low ..        41% Check
PLAT084_ALERT_3_C High wR2 Value (i.e. > 0.25) ...................       0.31 Report
PLAT230_ALERT_2_C Hirshfeld Test Diff for    C16      --C17      .        6.7 s.u.
PLAT241_ALERT_2_C High   'MainMol' Ueq as Compared to Neighbors of        C16 Check
PLAT250_ALERT_2_C Large U3/U1 Ratio for Average U(i,j) Tensor ....        2.1 Note
PLAT340_ALERT_3_C Low Bond Precision on  C-C Bonds ...............    0.00725 Ang.
PLAT352_ALERT_3_C Short  N-H (X0.87,N1.01A)  N10      - H10A     .       0.76 Ang.
PLAT416_ALERT_2_C Short Intra D-H..H-D       H8B      ..H10B     .       1.93 Ang.
                                                      x,y,z  =      1_555 Check
PLAT420_ALERT_2_C D-H Bond Without Acceptor  N3       --H3A      .     Please Check
PLAT420_ALERT_2_C D-H Bond Without Acceptor  N5       --H5A      .     Please Check
PLAT420_ALERT_2_C D-H Bond Without Acceptor  N8       --H8B      .     Please Check
PLAT420_ALERT_2_C D-H Bond Without Acceptor  N10      --H10B     .     Please Check
PLAT906_ALERT_3_C Large K Value in the Analysis of Variance ......      5.092 Check
PLAT911_ALERT_3_C Missing FCF Refl Between Thmin & STh/L=    0.600         17 Report
```

Alert level G

```
PLAT007_ALERT_5_G Number of Unrefined Donor-H Atoms ..............          4 Report
PLAT042_ALERT_1_G Calc. and Reported Moiety Formula Strings Differ     Please Check
PLAT045_ALERT_1_G Calculated and Reported Z Differ by a Factor ...       2.00 Check
PLAT063_ALERT_4_G Crystal Size Possibly too Large for Beam Size ..       0.63 mm
PLAT072_ALERT_2_G SHELXL First  Parameter in WGHT  Unusually Large       0.16 Report
PLAT912_ALERT_4_G Missing # of FCF Reflections Above STh/L=  0.600        141 Note
PLAT933_ALERT_2_G Number of OMIT Records in Embedded .res File ...          1 Note
PLAT941_ALERT_3_G Average HKL Measurement Multiplicity ...........        3.6 Low
PLAT978_ALERT_2_G Number C-C Bonds with Positive Residual Density.          0 Info
PLAT992_ALERT_5_G Repd & Actual _reflns_number_gt Values Differ by          2 Check
```

```
   0 ALERT level A = Most likely a serious problem - resolve or explain
   0 ALERT level B = A potentially serious problem, consider carefully
  15 ALERT level C = Check. Ensure it is not caused by an omission or oversight
  10 ALERT level G = General information/check it is not something unexpected

   2 ALERT type 1 CIF construction/syntax error, inconsistent or missing data
  11 ALERT type 2 Indicator that the structure model may be wrong or deficient
   8 ALERT type 3 Indicator that the structure quality may be low
   2 ALERT type 4 Improvement, methodology, query or suggestion
   2 ALERT type 5 Informative message, check
```

It is advisable to attempt to resolve as many as possible of the alerts in all categories. Often the minor alerts point to easily fixed oversights, errors and omissions in your CIF or refinement strategy, so attention to these fine details can be worthwhile. In order to resolve some of the more serious problems it may be necessary to carry out additional measurements or structure refinements. However, the purpose of your study may justify the reported deviations and the more serious of these should normally be commented upon in the discussion or experimental section of a paper or in the "special_details" fields of the CIF. checkCIF was carefully designed to identify outliers and unusual parameters, but every test has its limitations and alerts that are not important in a particular case may appear. Conversely, the absence of alerts does not guarantee there are no aspects of the results needing attention. It is up to the individual to critically assess their own results and, if necessary, seek expert advice.

Publication of your CIF in IUCr journals

A basic structural check has been run on your CIF. These basic checks will be run on all CIFs submitted for publication in IUCr journals (*Acta Crystallographica*, *Journal of Applied Crystallography*, *Journal of Synchrotron Radiation*); however, if you intend to submit to *Acta Crystallographica Section C* or *E* or *IUCrData*, you should make sure that full publication checks are run on the final version of your CIF prior to submission.

Publication of your CIF in other journals

Please refer to the *Notes for Authors* of the relevant journal for any special instructions relating to CIF submission.

PLATON version of 22/03/2021; check.def file version of 19/03/2021

Datablock 3 - ellipsoid plot

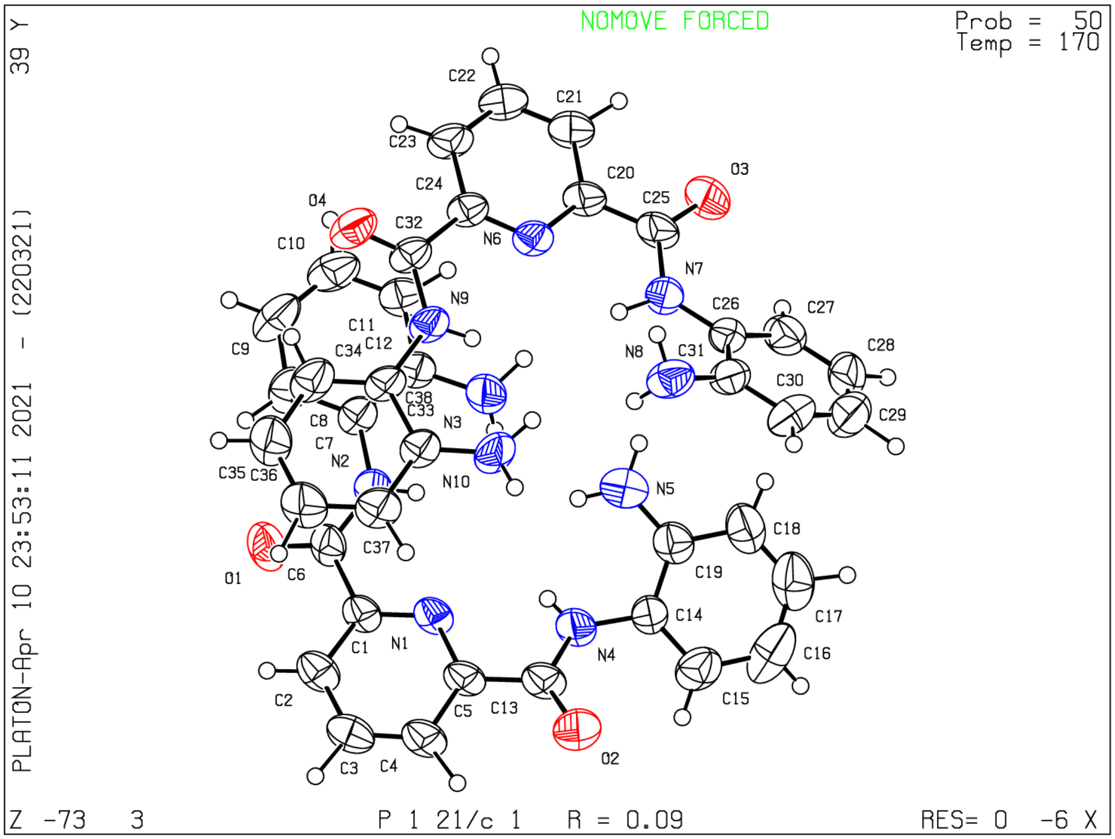

Supplement: Supplementary file 2 [file DataSheet4.PDF]
