# Supplementary figures and images for: Influence of Terminal Functionality on the Crystal Packing Behaviour and Cytotoxicity of Aromatic Oligoamides
Source: Front Chem. 2021 Jun 30;9:709161. doi: 10.3389/fchem.2021.709161 (PMC8277928; doi:10.3389/fchem.2021.709161)

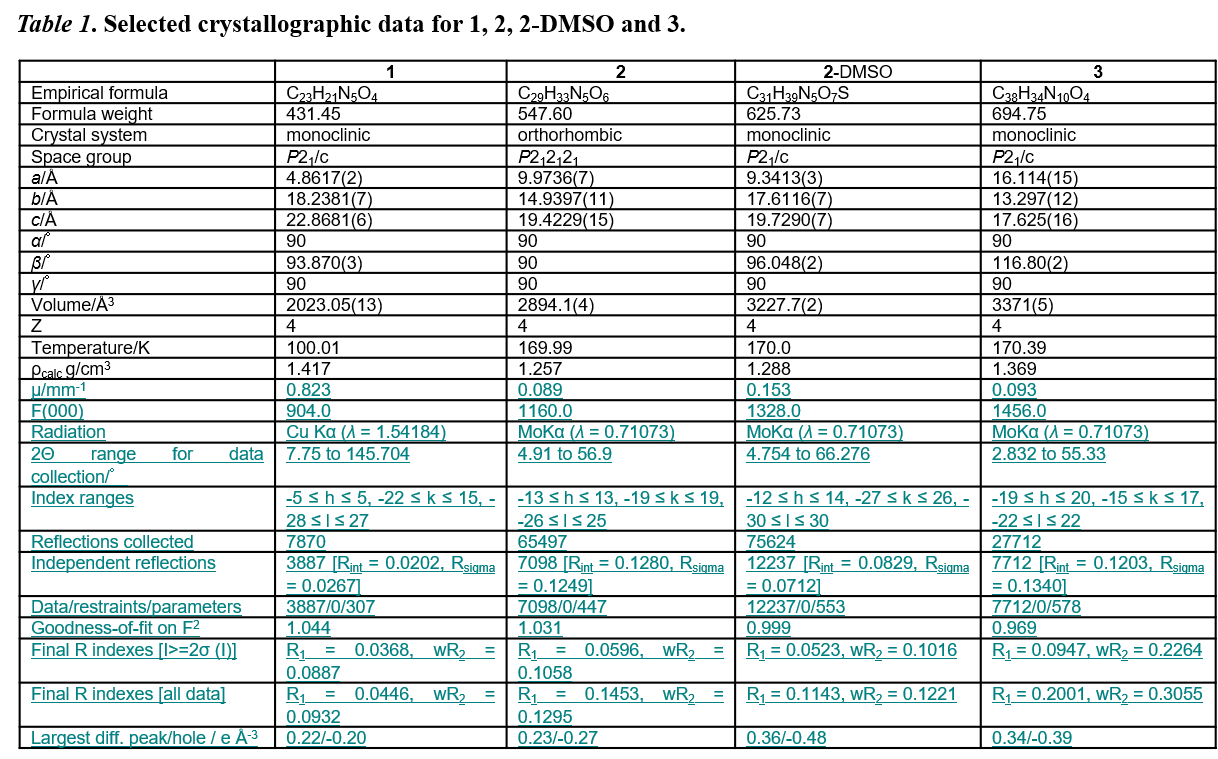

Supplement: Supplementary file 3 [file Image2.TIF]

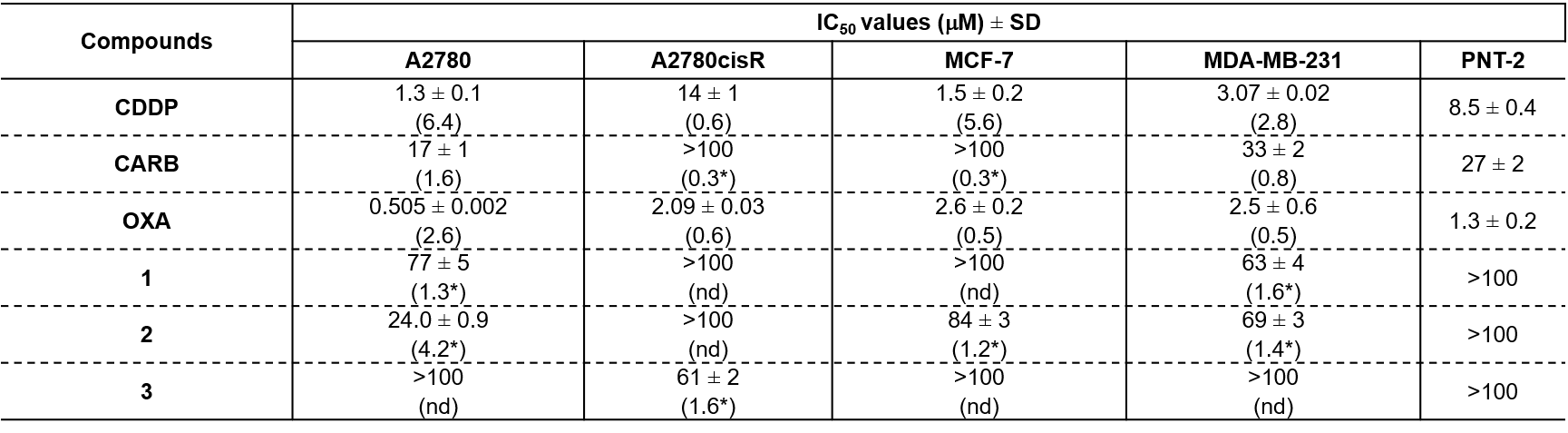

Supplement: Supplementary file 4 [file Image1.TIF]
