# Supplementary material for: Influence of Terminal Functionality on the Crystal Packing Behaviour and Cytotoxicity of Aromatic Oligoamides
Source: Front Chem. 2021 Jun 30;9:709161. doi: 10.3389/fchem.2021.709161 (PMC8277928; doi:10.3389/fchem.2021.709161)

checkCIF/PLATON report

Structure factors have been supplied for datablock(s) 2-DMSO

THIS REPORT IS FOR GUIDANCE ONLY. IF USED AS PART OF A REVIEW PROCEDURE FOR PUBLICATION, IT SHOULD NOT REPLACE THE EXPERTISE OF AN EXPERIENCED CRYSTALLOGRAPHIC REFEREE.

No syntax errors found. CIF dictionary Interpreting this report

Datablock: 2-DMSO

```
Bond precision:  C-C = 0.0020 A          Wavelength=0.71073

Cell:            a=9.3413(3)     b=17.6116(7)       c=19.7290(7)
                 alpha=90        beta=96.048(2)     gamma=90
Temperature:     170 K

                Calculated                 Reported
Volume          3227.7(2)                  3227.7(2)
Space group     P 21/c                     P 1 21/c 1
Hall group      -P 2ybc                    -P 2ybc
Moiety formula  C29 H33 N5 O6, C2 H6 O S   C2 H6 O S, C29 H33 N5 O6
Sum formula     C31 H39 N5 O7 S            C31 H39 N5 O7 S
Mr              625.73                     625.73
Dx,g cm-3       1.288                      1.288
Z               4                          4
Mu (mm-1)       0.153                      0.153
F000            1328.0                     1328.0
F000'           1329.09
h,k,lmax        14,27,30                   14,27,30
Nref            12304                      12239
Tmin,Tmax       0.978,0.985                0.674,0.747
Tmin'           0.944

Correction method= # Reported T Limits: Tmin=0.674 Tmax=0.747
AbsCorr = MULTI-SCAN

Data completeness= 0.995          Theta(max)= 33.138

R(reflections)= 0.0525( 7084)     wR2(reflections)= 0.1254( 12239)

S = 0.999                 Npar= 553
```

The following ALERTS were generated. Each ALERT has the format
test-name_ALERT_alert-type_alert-level.
Click on the hyperlinks for more details of the test.

Alert level C

```
PLAT905_ALERT_3_C Negative K value in the Analysis of Variance ...     -0.307 Report
```

Alert level G

```
PLAT042_ALERT_1_G Calc. and Reported Moiety Formula Strings Differ     Please Check
PLAT910_ALERT_3_G Missing # of FCF Reflection(s) Below Theta(Min).          4 Note
PLAT912_ALERT_4_G Missing # of FCF Reflections Above STh/L=  0.600         62 Note
PLAT933_ALERT_2_G Number of OMIT Records in Embedded .res File ...          3 Note
PLAT978_ALERT_2_G Number C-C Bonds with Positive Residual Density.         12 Info
```

```
   0 ALERT level A = Most likely a serious problem - resolve or explain
   0 ALERT level B = A potentially serious problem, consider carefully
   1 ALERT level C = Check. Ensure it is not caused by an omission or oversight
   5 ALERT level G = General information/check it is not something unexpected

   1 ALERT type 1 CIF construction/syntax error, inconsistent or missing data
   2 ALERT type 2 Indicator that the structure model may be wrong or deficient
   2 ALERT type 3 Indicator that the structure quality may be low
   1 ALERT type 4 Improvement, methodology, query or suggestion
   0 ALERT type 5 Informative message, check
```

It is advisable to attempt to resolve as many as possible of the alerts in all categories. Often the minor alerts point to easily fixed oversights, errors and omissions in your CIF or refinement strategy, so attention to these fine details can be worthwhile. In order to resolve some of the more serious problems it may be necessary to carry out additional measurements or structure refinements. However, the purpose of your study may justify the reported deviations and the more serious of these should normally be commented upon in the discussion or experimental section of a paper or in the "special_details" fields of the CIF. checkCIF was carefully designed to identify outliers and unusual parameters, but every test has its limitations and alerts that are not important in a particular case may appear. Conversely, the absence of alerts does not guarantee there are no aspects of the results needing attention. It is up to the individual to critically assess their own results and, if necessary, seek expert advice.

Publication of your CIF in IUCr journals

A basic structural check has been run on your CIF. These basic checks will be run on all CIFs submitted for publication in IUCr journals (*Acta Crystallographica*, *Journal of Applied Crystallography*, *Journal of Synchrotron Radiation*); however, if you intend to submit to *Acta Crystallographica Section C* or *E* or *IUCrData*, you should make sure that full publication checks are run on the final version of your CIF prior to submission.

Publication of your CIF in other journals

Please refer to the *Notes for Authors* of the relevant journal for any special instructions relating to CIF submission.

PLATON version of 22/03/2021; check.def file version of 19/03/2021

Datablock 2-DMSO - ellipsoid plot

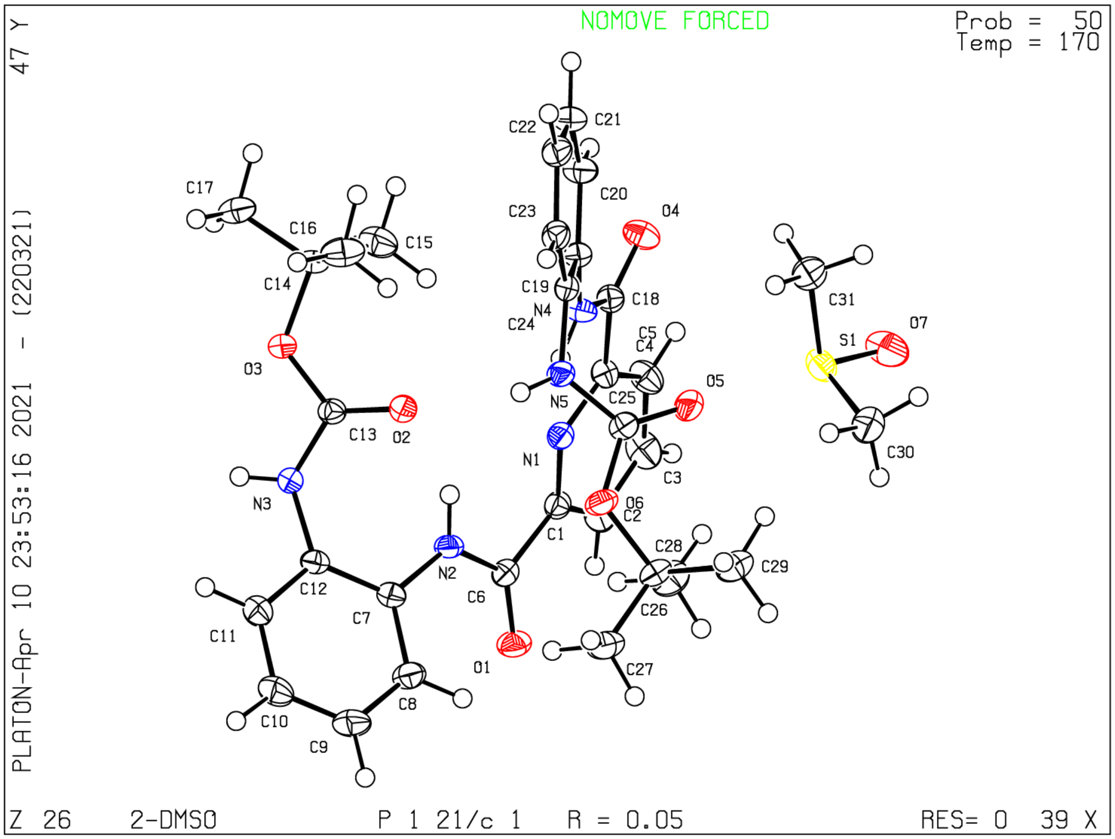

Supplement: Supplementary file 5 [file DataSheet3.PDF]
