# Supplementary material for: Influence of Terminal Functionality on the Crystal Packing Behaviour and Cytotoxicity of Aromatic Oligoamides
Source: Front Chem. 2021 Jun 30;9:709161. doi: 10.3389/fchem.2021.709161 (PMC8277928; doi:10.3389/fchem.2021.709161)

checkCIF/PLATON report

Structure factors have been supplied for datablock(s) 1

THIS REPORT IS FOR GUIDANCE ONLY. IF USED AS PART OF A REVIEW PROCEDURE FOR PUBLICATION, IT SHOULD NOT REPLACE THE EXPERTISE OF AN EXPERIENCED CRYSTALLOGRAPHIC REFEREE.

No syntax errors found.   CIF dictionary   Interpreting this report

Datablock: 1

```
Bond precision:  C-C = 0.0020 A          Wavelength=1.54184

Cell:            a=4.8617(2)     b=18.2381(7)       c=22.8681(6)
                 alpha=90        beta=93.870(3)     gamma=90
Temperature:     100 K

                Calculated                Reported
Volume          2023.05(13)               2023.05(13)
Space group     P 21/c                    P 1 21/c 1
Hall group      -P 2ybc                   -P 2ybc
Moiety formula  C23 H21 N5 O4             C23 H21 N5 O4
Sum formula     C23 H21 N5 O4             C23 H21 N5 O4
Mr              431.45                    431.45
Dx,g cm-3       1.417                     1.417
Z               4                         4
Mu (mm-1)       0.823                     0.823
F000            904.0                     904.0
F000'           906.88
h,k,lmax        6,22,28                   5,22,28
Nref            4018                      3887
Tmin,Tmax       0.954,0.968               0.784,1.000
Tmin'           0.835

Correction method= # Reported T Limits: Tmin=0.784 Tmax=1.000
AbsCorr = GAUSSIAN

Data completeness= 0.967          Theta(max)= 72.852

R(reflections)= 0.0368( 3357)     wR2(reflections)= 0.0932( 3887)

S = 1.044                  Npar= 307
```

The following ALERTS were generated. Each ALERT has the format
test-name_ALERT_alert-type_alert-level.
Click on the hyperlinks for more details of the test.

Alert level C

```
PLAT411_ALERT_2_C Short Inter H...H Contact  H2       ..H2       .       2.00 Ang.
                                              -1-x,1-y,1-z  =      3_466 Check
PLAT911_ALERT_3_C Missing FCF Refl Between Thmin & STh/L=    0.600          2 Report
```

Alert level G

```
PLAT380_ALERT_4_G Incorrectly? Oriented X(sp2)-Methyl Moiety .....        C23 Check
PLAT432_ALERT_2_G Short Inter X...Y Contact  C15      ..C21              3.13 Ang.
                                                  -1+x,y,z  =      1_455 Check
PLAT910_ALERT_3_G Missing # of FCF Reflection(s) Below Theta(Min).          1 Note
PLAT912_ALERT_4_G Missing # of FCF Reflections Above STh/L=  0.600        119 Note
PLAT941_ALERT_3_G Average HKL Measurement Multiplicity ..........        2.0 Low
PLAT978_ALERT_2_G Number C-C Bonds with Positive Residual Density.         15 Info
```

---

0 **ALERT level A** = Most likely a serious problem - resolve or explain
0 **ALERT level B** = A potentially serious problem, consider carefully
2 **ALERT level C** = Check. Ensure it is not caused by an omission or oversight
6 **ALERT level G** = General information/check it is not something unexpected

0 ALERT type 1 CIF construction/syntax error, inconsistent or missing data
3 ALERT type 2 Indicator that the structure model may be wrong or deficient
3 ALERT type 3 Indicator that the structure quality may be low
2 ALERT type 4 Improvement, methodology, query or suggestion
0 ALERT type 5 Informative message, check

It is advisable to attempt to resolve as many as possible of the alerts in all categories. Often the minor alerts point to easily fixed oversights, errors and omissions in your CIF or refinement strategy, so attention to these fine details can be worthwhile. In order to resolve some of the more serious problems it may be necessary to carry out additional measurements or structure refinements. However, the purpose of your study may justify the reported deviations and the more serious of these should normally be commented upon in the discussion or experimental section of a paper or in the "special_details" fields of the CIF. checkCIF was carefully designed to identify outliers and unusual parameters, but every test has its limitations and alerts that are not important in a particular case may appear. Conversely, the absence of alerts does not guarantee there are no aspects of the results needing attention. It is up to the individual to critically assess their own results and, if necessary, seek expert advice.

Publication of your CIF in IUCr journals

A basic structural check has been run on your CIF. These basic checks will be run on all CIFs submitted for publication in IUCr journals (*Acta Crystallographica*, *Journal of Applied Crystallography*, *Journal of Synchrotron Radiation*); however, if you intend to submit to *Acta Crystallographica Section C* or *E* or *IUCrData*, you should make sure that full publication checks are run on the final version of your CIF prior to submission.

Publication of your CIF in other journals

Please refer to the *Notes for Authors* of the relevant journal for any special instructions relating to CIF submission.

PLATON version of 22/03/2021; check.def file version of 19/03/2021

Datablock 1 - ellipsoid plot

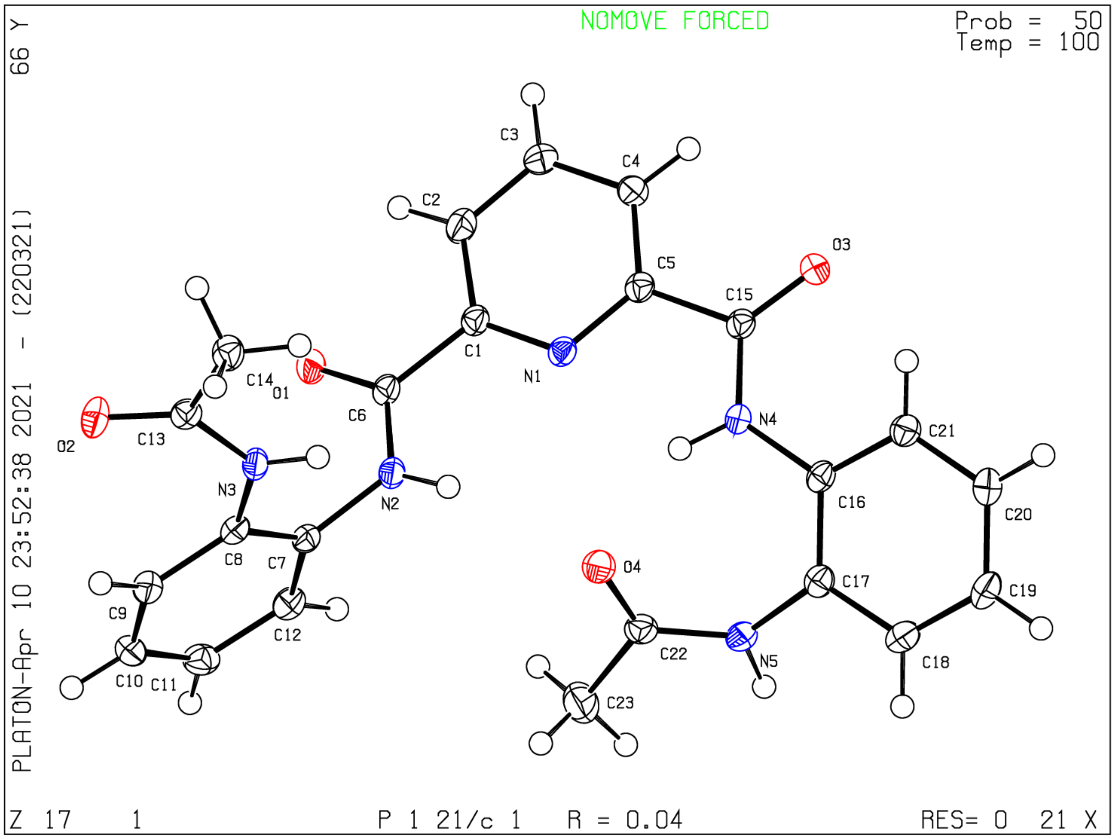

Supplement: Supplementary file 6 [file DataSheet1.PDF]
