# Supplementary material for: Influence of Terminal Functionality on the Crystal Packing Behaviour and Cytotoxicity of Aromatic Oligoamides
Source: Front Chem. 2021 Jun 30;9:709161. doi: 10.3389/fchem.2021.709161 (PMC8277928; doi:10.3389/fchem.2021.709161)
Supplement: Supplementary file 7 [file DataSheet5.pdf]

Supporting Information

Influence of Terminal Functionality on the Crystal Packing Behaviour and Cytotoxicity of Aromatic Oligoamides

Pierre Delfosse,[1] Colin C. Seaton,[1] Louise Male,[2] Rianne M. Lord,[1,3*] and Sarah J. Pike[1,2*]

[1]School of Chemistry and Biosciences, University of Bradford, Bradford, BD7 1DP, U.K.

[2]School of Chemistry, University of Birmingham, Edgbaston, Birmingham, B15 2TT, U. K.

[3]School of Chemistry, University of East Anglia, Norwich Research Park, Norwich, NR4 7TJ, U.K.

Email: r.lord@uea.ac.uk, s.pike@bham.ac.uk

Contents *S2*

General Experimental Details *S3*

Crystallographic Details *S3*

Cell Culture *S5*

Statistical Analysis *S6*

Synthetic Details *S7*

Crystal data and structural refinement of 1 *S10*

Crystal data and structural refinement of 2 *S11*

Crystal data and structural refinement of 2-DMSO *S12*

Crystal data and structural refinement of 3 *S13*

Solid State Analysis of 1 *S22*

Solid State Analysis of 2 *S25*

Solid State Analysis of 2-DMSO *S29*

Solid State Analysis of 3 *S31*

Appendix: ^{1}H and ^{13}C NMR spectra *S35*

References *S38*

Experimental Section

General Experimental Details

All NMR spectroscopy was carried out on a Bruker Advance 400 FT NMR spectrometer using the residual solvent as the internal standard. All the chemical shifts (δ) are quoted in ppm and coupling constants are given in Hz and are rounded to 0.1 Hz. Melting points were obtained on GallenKemp and are uncorrected. Infrared spectroscopy was carried out on a Perkin Elmer 100 FT-IR instrument fitted with an ATR detector. Mass spectrometry was recorded on a Waters Micromass Quattro Ultima quadrupole mass spectrometer at the Bradford Analytical Centre. 2,6-dipyridinecarbonyl dichloride and trifluoroacetic acid were purchased from Acros Organics. Pyridine was purchased from Sigma Aldrich. Dichloromethane, petroleum ether, dimethylsulfoxide and chloroform were purchased from Fisher. All the reactions were conducted under nitrogen atmosphere. Petrol refers to the fraction of light petroleum ether boiling between 40 and 60 °C. All chemicals were used as received unless otherwise stated. The following abbreviations are employed: aq. = aqueous, Ar = aromatic, br = broad, Boc = *tert*-butyloxycarbonyl, calc. = calculated, CARB = carboplatin, CDDP = cisplatin, d = doublet, DMF = dimethylformamide, DMSO = dimethylsulfoxide, eq. = equivalent(s), Et = ethyl, h = hour(s), Hz = Hertz, IC_{50} = half maximal inhibitory concentration, IR = infrared, m = multiplet, Me = methyl, m.p. = melting point, MTT = 3-(4,5-dimethylthiazol-2-yl)-2,5-diphenyltetrazolium bromide, OXA = oxaplatin, pet. = petroleum, rt = room temperature, s = singlet, SD = standard deviation, SI = selectivity index, t = triplet, TFA = trifluoroacetic acid and TNBC = triplet negative breast cancer. *N*-(2-aminophenyl)acetamide and *tert*-butyl-*N*-(2-aminophenyl)carbamate were prepared according to literature procedures.[1,2]

Crystallographic Details

The dataset for **1** was measured on an Agilent SuperNova diffractometer using an Atlas detector. The data collection was driven and processed, and an absorption correction was applied using CrysAlisPro.[3] The datasets for **2**, **2**-DMSO and **3** were measured on a Bruker APEX-II CCD diffractometer. The data collections were processed by SAINT[4] and absorption corrections were applied using SADABS.[5] The structures of **2**, **2**-DMSO and **3** were solved using ShelXS[6] while that of **1** was solved using ShelXT.[7] All structures were refined by a full-matrix least-squares procedure on F2 in ShelXL.[8] Figures and reports were produced using OLEX2.[9] All non-hydrogen atoms were refined with anisotropic displacement parameters.

Crystal structures were visualised using Mercury. The X-ray data for **1**, **2**, **2**-DMSO and **3** have been deposited with the Cambridge Crystallographic Data Centre: **1**: 2081178, **2**: 2081175, **2**-DMSO: 2081176 and **3**: 2081174.

Crystal data for **1**: C23H21N5O4 (M =431.45 g/mol): monoclinic, space group $P2_1/c$ (no. 14), a = 4.8617(2) Å, b = 18.2381(7) Å, c = 22.8681(6) Å, β = 93.870(3)°, V = 2023.05(13) $Å^3$, Z = 4, T = 100.01(10) K, μ(Cu Kα) = 0.823 mm^{-1}, D_{calc} = 1.417 g/cm^3, 7870 reflections measured (7.75° ≤ 2Θ ≤ 145.704°), 3887 unique (R_{int} = 0.0202, R_{sigma} = 0.0267) which were used in all calculations. The final R1 was 0.0368 (I > 2σ(I)) and wR2 was 0.0932 (all data).

The hydrogen atoms bonded to N(2), N(3), N(4) and N(5) were located in the electron density and freely refined. The remaining hydrogen atoms were fixed as riding models with the isotropic thermal parameters (Uiso) based on the Ueq of the parent atom.

Crystal data for **2**: C29H33N5O6 (M =547.60 g/mol): orthorhombic, space group $P2_12_12_1$ (no. 19), a = 9.9736(7) Å, b = 14.9397(11) Å, c = 19.4229(15) Å, V = 2894.1(4) $Å^3$, Z = 4, T = 169.99 K, μ(MoKα) = 0.089 mm^{-1}, D_{calc} = 1.257 g/cm^3, 65497 reflections measured (4.91° ≤ 2Θ ≤ 56.9°), 7098 unique (R_{int} = 0.1280, R_{sigma} = 0.1249) which were used in all calculations. The final R1 was 0.0596 (I > 2σ(I)) and wR2 was 0.1295 (all data).

The hydrogen atoms bonded to N(2), N(3), N(4), N(5), C(3), C(11), C(27) and C(28) were fixed as riding models with the isotropic thermal parameters (Uiso) based on the Ueq of the parent atom. The remaining hydrogen atoms were located in the electron density and freely refined.

Crystal data for **2**-DMSO: C31H39N5O7S (M =625.73 g/mol): monoclinic, space group $P2_1/c$ (no. 14), a = 9.3413(3) Å, b = 17.6116(7) Å, c = 19.7290(7) Å, β = 96.048(2)°, V = 3227.7(2) $Å^3$, Z = 4, T = 170.0 K, μ(MoKα) = 0.153 mm^{-1}, D_{calc} = 1.288 g/cm^3, 75632 reflections measured (4.754° ≤ 2Θ ≤ 66.276°), 12239 unique (R_{int} = 0.0829, R_{sigma} = 0.0712) which were used in all calculations. The final R1 was 0.0525 (I > 2σ(I)) and wR2 was 0.1254 (all data).

The structure contains a molecule of DMSO. The hydrogen atoms were located in the electron density and freely refined.

Crystal data for **3**: C38H34N10O4 (M =694.75 g/mol): monoclinic, space group $P2_1/c$ (no. 14), a = 16.114(15) Å, b = 13.297(12) Å, c = 17.625(16) Å, β = 116.80(2)°, V = 3371(5) $Å^3$, Z = 4,

T = 170.39 K, $\mu(MoK\alpha) = 0.093\ mm^{-1}$, $D_{calc} = 1.369\ g/cm^3$, 27712 reflections measured ($2.832° \leq 2\Theta \leq 55.33°$), 7712 unique ($R_{int} = 0.1203$, $R_{sigma} = 0.1340$) which were used in all calculations. The final R1 was 0.0947 ($I > 2\sigma(I)$) and wR2 was 0.3055 (all data).

The hydrogen atoms bonded to N(5), N(8), C(15), C(27) and C(30) were fixed as riding models with the isotropic thermal parameters (Uiso) based on the Ueq of the parent atom. The remaining hydrogen atoms were located in the electron density and freely refined.

Cell culture

In vitro chemosensitivity assays were performed against human ovarian carcinoma (A2780), cisplatin-resistant human ovarian carcinoma (A2780cisR) and human breast adenocarcinomas (MCF-7 and MDA-MB-231). Additionally, growth inhibitory effects were also tested against non-malignant prostate cell line immortalised with SV40, PNT-2. All cell lines were provided by the Institute of Cancer Therapeutics, University of Bradford and were routinely maintained as monolayer cultures in RPMI 1640 media supplemented with 10% foetal calf serum, sodium pyruvate (1 mM) and L-glutamine (2 mM). All assays were conducted in 96-well round bottom plates, with control lanes for media and 100% cell growth. Cell concentrations of 1 x 10^4 cells/mL were used, and 100 µL (or 100 µL media in control lane 1) of cell suspension were incubated for 24 h at 37 °C and 5% CO_2 prior to drug exposure. Compounds **1**-**3**, cisplatin (**CDDP**), oxaliplatin (**OXA**) and carboplatin (**CARB**) were all dissolved in DMSO to provide 100 mM stock solutions, which were further diluted with complete media to provide a range of final concentrations. After 24 h, 100 µL of the drug/media solutions were added to the plates (columns 3-12 drug concentrations, with 100 µL media in the control lanes), and then the plates incubated for 96 h at 37°C and 5% CO_2. All drug solutions were added to the plates so that the final DMSO concentrations were <0.1% (v/v) in all cases. After 96 h, 3-(4,5-dimethylthiazol-2-yl)-2,5-diphenyltetrazolium bromide (MTT) (20 µL, 5 mg/mL) was added to each well and incubated for 3 h at 37 °C and 5% CO_2. All solutions were then removed via pipette, 150 µL DMSO added to each well and the formazan crystals mixed thoroughly. A Thermo Scientific Multiscan EX microplate photometer was used to measure the absorbance of each well at 540 nm. Percentage cell viabilities were determined on a logarithmic scale, and the half maximal inhibitory concentration (IC_{50}) determined from a plot of % cell survival *versus* concentration (µM). Each of the experiments was performed as duplicate technical repeats and triplicate experimental repeats, with mean values as the IC_{50} ± Standard Deviation (SD).

Statistical Analysis

A two-tailed ANOVA t-test has been conducted using Graph Pad Prism 8 and used to compare all chemosensitivity data: probability values $p < 0.05$ are considered significant.

Synthetic Details

1

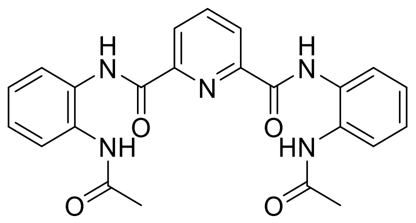

To a solution of 2,6-dipyridinecarbonyl dichloride (0.20 g, 0.98 mmol) in dry CH_2Cl_2 (2 mL) was added *N*-(2-aminophenyl)acetamide (0.30 g, 2.0 mmol) in dry CH_2Cl_2 (6 mL) in a dropwise manner and the resultant solution was left stirring at ambient temperature for 18 h. After this time, the white precipitate that had formed was isolated by filtration and the resultant white solid was washed with H_2O (100 mL) and dried under vacuum to yield the desired product as white solid. Single crystals suitable for X-ray diffraction analysis were grown through the vapour diffusion of Et_2O into a DMF solution (0.18 g, 42%). m.p.: 264 °C; ^{1}H NMR (400 MHz, DMSO-d_6): δ 10.93 (s, 2H, N*H*Ac), 9.79 (s, 2H, N*H*), 8.41-8.39 (m, 2H, Ar*H*), 8.35-8.31 (m, 1H, Ar*H*), 7.77-7.74 (m, 2H, Ar*H*), 7.58-7.56 (m, 2H, Ar*H*), 7.27-7.25 (m, 4H, ArH), 1.91 (s, 6H, 2 × C*H*$_3$); ^{13}C NMR (100 MHz, DMSO-d_6): δ 169.4 (CO), 161.6 (CO), 148.6 (Ar), 140.2 (Ar), 131.6 (Ar), 129.6 (Ar), 125.8 (Ar), 125.6 (Ar), 125.0 (2C, Ar), 124.5 (Ar), 23.3 (CH_3); IR (solid) ν_{max} 3244 (m), 3073 (m), 1664 (s), 1645 (s), 1608 (s), 1593 (s), 1526 (s), 1504 (s); LR-ESIMS: m/z = 432 $[M+H]^+$; HR-ESIMS: *m/z* = 432.1670 (calc. for $C_{23}H_{22}O_4N_5$, 432.17666).

2

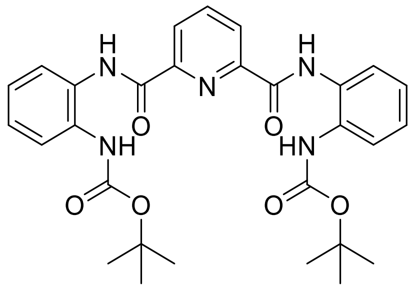

To a solution of 2,6-pyridinedicarbonyl dichloride (0.60 g, 2.9 mmol), pyridine (0.60 mL, 7.3 mmol) in dry CH_2Cl_2 (50 mL) was added a solution of *tert*-butyl-*N*-(2-aminophenyl)carbamate (1.3 g, 6.0 mmol) in dry CH_2Cl_2 (18 mL) in a dropwise manner and the resultant solution was stirred at ambient temperature for 18 h. After this time, the excess solvent was removed under reduced pressure and the resultant residue was subjected to flash column chromatography (Pet. ether/EtOAc; 2/1) to yield the desired product as a white solid. Single crystals suitable for X-ray diffraction analysis were grown through the slow evaporation of chloroform (0.25 g, 15%). m.p.: 215-217 °C; ^{1}H NMR (400 MHz, $CDCl_3$): δ 10.73 (s, br, 2H, N*H*Boc), 8.52 (d, 2H, ^{3}J = 7.7 Hz, Ar*H*), 8.16 (t, 1H, ^{3}J = 7.7 Hz, Ar*H*), 7.94 (s, br, 2H, N*H*), 7.37 (d, 2H, ^{3}J = 7.9 Hz, Ar*H*), 7.21-7.17 (m, 6H, Ar*H*), 1.24 (s, 18H, 2 × (CH_3)$_3$); ^{13}C NMR (100 MHz, $CDCl_3$/DMSO-d_6 9/1): δ 162.1 (CO), 153.7 (CO), 148.5 (Ar), 139.1 (Ar), 131.7 (Ar), 129.0 (Ar), 126.3 (Ar), 125.4 (Ar), 125.2 (Ar), 124.6 (Ar), 124.4 (Ar), 80.2 (αC), 27.9 (CH_3); IR (solid) ν_{max} 3342 (m), 3268 (m), 3042 (w), 2967 (w), 2972 (w), 2922 (m), 2847 (m), 1713 (s), 1699 (s), 1668 (s), 1599 (m), 1532 (s), 1513 (s); LR-ESIMS: m/z = 548 $[M+H]^+$; HR-ESIMS: *m/z* = 548.2503 (calc. for $C_{29}H_{34}O_6N_5$, 548.2504); Elemental Analysis: Anal. Found: C: 63.36, H: 5.89, N: 12.63%. Anal. Calculated: C: 63.61, H: 6.07, N: 12.79%.

3

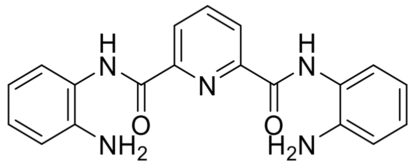

To a solution of **2** (0.07 g, 0.13 mmol) in CH_2Cl_2 (4 mL) was added TFA (0.28 mL, 3.7 mmol) and the resultant solution was stirred at ambient temperature for 18 h. After this time, aqueous Na_2CO_3 (10 mL) was added to the solution, and the aqueous phase was extracted with CH_2Cl_2 (4 × 20 mL). The organic layers were combined and washed with H_2O (2 × 20 mL), dried over $MgSO_4$, filtered and the excess solvent was removed under reduced pressure to yield the desired product as a yellow solid. Single crystals suitable for X-ray diffraction analysis were grown through the slow evaporation of chloroform (0.030 g, 67%). ^{1}H NMR (400 MHz, $CDCl_3$): δ 9.73 (s, 2H, N*H*), 8.50 (d, 2H J = 7.8 Hz, Ar*H*), 8.15 (t, 1H, J = 7.7 Hz, ArH), 7.62 (dd, 2H, ^{3}J = 7.9 Hz, ^{4}J = 1.4 Hz, Ar*H*), 7.11 (dt, 2H, ^{3}J = 7.7 Hz, ^{4}J = 1.4 Hz, Ar*H*), 6.94-6.88 (m, 4H, Ar*H*), 3.90 (s, 4H, 2 × NH_2); ^{13}C NMR (100 MHz, $CDCl_3$): δ 161.2 (CO), 148.7 (Ar), 139.6 (Ar), 139.5 (Ar), 126.9 (Ar), 125.6 (Ar), 124.8 (Ar), 124.2 (Ar), 120.4 (Ar), 118.8 (Ar); IR (solid) ν_{max} 3384 (m), 3330 (m), 3221 (m), 2911 (w), 1668 (s), 1621 (m), 1592 (s), 1568 (m), 1521 (s), 1491 (s); LR-ESIMS: *m/z* = 348 $[M+H]^+$; HR-ESIMS: m/z = 348.1453 (calc. for $C_{19}H_{18}O_2N_5$, 348.1455).

Crystal Data and Structural Refinement

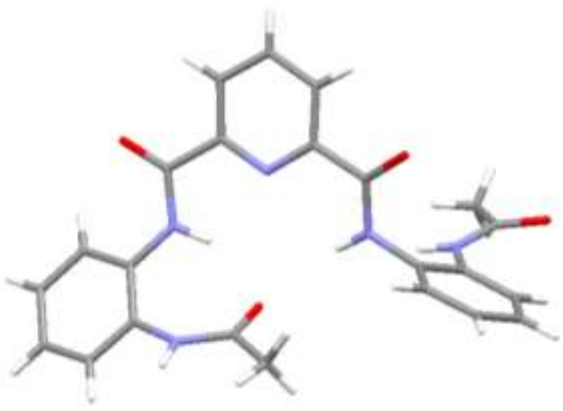

Table 2 Crystal data and structure refinement for 1.

Identification code	**1**
Empirical formula	$C_{23}H_{21}N_5O_4$
Formula weight	431.45
Temperature/K	100.01(10)
Crystal system	monoclinic
Space group	$P2_1/c$
a/Å	4.8617(2)
b/Å	18.2381(7)
c/Å	22.8681(6)
α/°	90
β/°	93.870(3)
γ/°	90
Volume/Å^3	2023.05(13)
Z	4
ρ_{calc}g/cm^3	1.417
μ/mm^{-1}	0.823
F(000)	904.0
Crystal size/mm^3	0.219 × 0.048 × 0.039
Radiation	Cu Kα (λ = 1.54184)
2Θ range for data collection/°	7.75 to 145.704
Index ranges	$-5 \leq h \leq 5$, $-22 \leq k \leq 15$, $-28 \leq l \leq 27$
Reflections collected	7870
Independent reflections	3887 [R_{int} = 0.0202, R_{sigma} = 0.0267]
Data/restraints/parameters	3887/0/307
Goodness-of-fit on F^2	1.044
Final R indexes [I>=2σ (I)]	R_1 = 0.0368, wR_2 = 0.0887
Final R indexes [all data]	R_1 = 0.0446, wR_2 = 0.0932
Largest diff. peak/hole / e Å^{-3}	0.22/-0.20

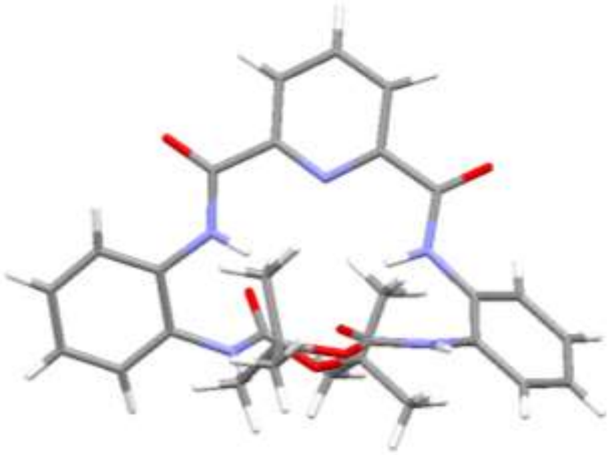

Table 3 Crystal data and structure refinement for **2**.

Identification code	**2**
Empirical formula	$C_{29}H_{33}N_5O_6$
Formula weight	547.60
Temperature/K	169.99
Crystal system	orthorhombic
Space group	$P2_12_12_1$
a/Å	9.9736(7)
b/Å	14.9397(11)
c/Å	19.4229(15)
α/°	90
β/°	90
γ/°	90
Volume/Å^3	2894.1(4)
Z	4
ρ_{calc}g/cm^3	1.257
μ/mm^{-1}	0.089
F(000)	1160.0
Crystal size/mm^3	0.96 × 0.1 × 0.1
Radiation	MoKα (λ = 0.71073)
2Θ range for data collection/°	4.91 to 56.9
Index ranges	$-13 \leq h \leq 13$, $-19 \leq k \leq 19$, $-26 \leq l \leq 25$
Reflections collected	65497
Independent reflections	7098 [R_{int} = 0.1280, R_{sigma} = 0.1249]
Data/restraints/parameters	7098/0/447
Goodness-of-fit on F^2	1.031
Final R indexes [I>=2σ (I)]	R_1 = 0.0596, wR_2 = 0.1058
Final R indexes [all data]	R_1 = 0.1453, wR_2 = 0.1295
Largest diff. peak/hole / e Å^{-3}	0.23/-0.27
Flack parameter	0.5(7)

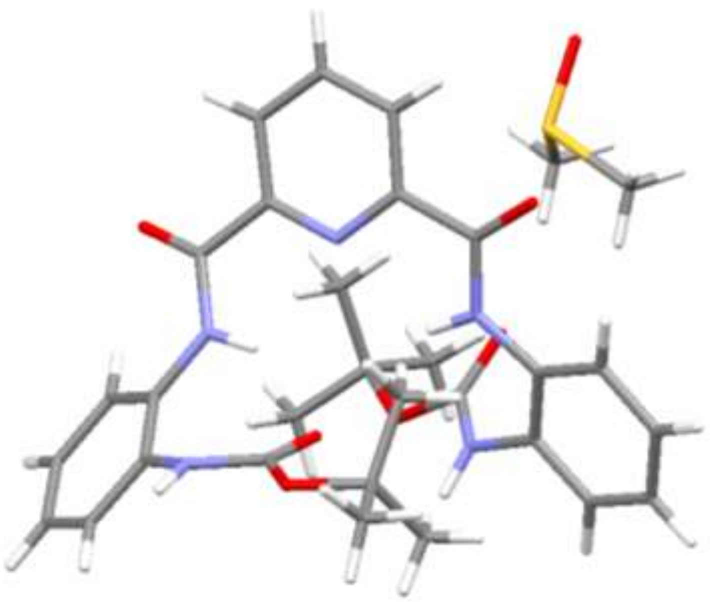

Table 4 Crystal data and structure refinement for **2**-DMSO.

Identification code	**2**-DMSO
Empirical formula	$C_{31}H_{39}N_5O_7S$
Formula weight	625.73
Temperature/K	170.0
Crystal system	monoclinic
Space group	$P2_1/c$
a/Å	9.3413(3)
b/Å	17.6116(7)
c/Å	19.7290(7)
α/°	90
β/°	96.048(2)
γ/°	90
Volume/Å^3	3227.7(2)
Z	4
ρ_{calc}g/cm^3	1.288
μ/mm^{-1}	0.153
F(000)	1328.0
Crystal size/mm^3	0.38 × 0.12 × 0.1
Radiation	MoKα (λ = 0.71073)
2Θ range for data collection/°	4.754 to 66.276
Index ranges	$-12 \leq h \leq 14$, $-27 \leq k \leq 26$, $-30 \leq l \leq 30$
Reflections collected	75624
Independent reflections	12237 [R_{int} = 0.0829, R_{sigma} = 0.0712]
Data/restraints/parameters	12237/0/553
Goodness-of-fit on F^2	0.999
Final R indexes [I>=2σ (I)]	R_1 = 0.0523, wR_2 = 0.1016
Final R indexes [all data]	R_1 = 0.1143, wR_2 = 0.1221
Largest diff. peak/hole / e Å^{-3}	0.36/-0.48

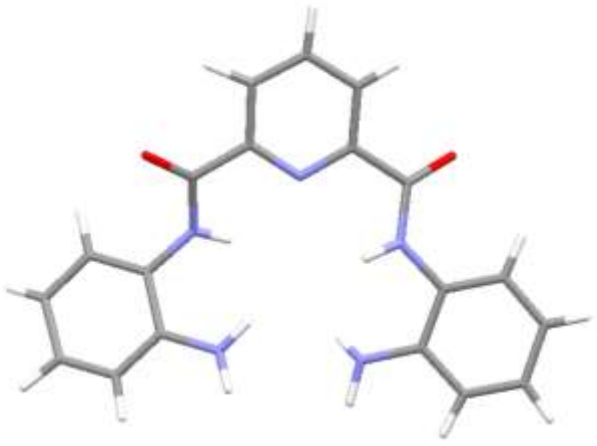

Table 5 Crystal data and structure refinement for 3

Identification code	**3**
Empirical formula	$C_{38}H_{34}N_{10}O_4$
Formula weight	694.75
Temperature/K	170.39
Crystal system	monoclinic
Space group	$P2_1/c$
a/Å	16.114(15)
b/Å	13.297(12)
c/Å	17.625(16)
α/°	90
β/°	116.80(2)
γ/°	90
Volume/Å^3	3371(5)
Z	4
ρ_{calc}g/cm^3	1.369
μ/mm^{-1}	0.093
F(000)	1456.0
Crystal size/mm^3	0.63 × 0.5 × 0.33
Radiation	MoKα (λ = 0.71073)
2Θ range for data collection/°	2.832 to 55.33
Index ranges	-19 ≤ h ≤ 20, -15 ≤ k ≤ 17, -22 ≤ l ≤ 22
Reflections collected	27712
Independent reflections	7712 [R_{int} = 0.1203, R_{sigma} = 0.1340]
Data/restraints/parameters	7712/0/578
Goodness-of-fit on F^2	0.969
Final R indexes [I>=2σ (I)]	R_1 = 0.0947, wR_2 = 0.2264
Final R indexes [all data]	R_1 = 0.2001, wR_2 = 0.3055
Largest diff. peak/hole / e Å^{-3}	0.34/-0.39

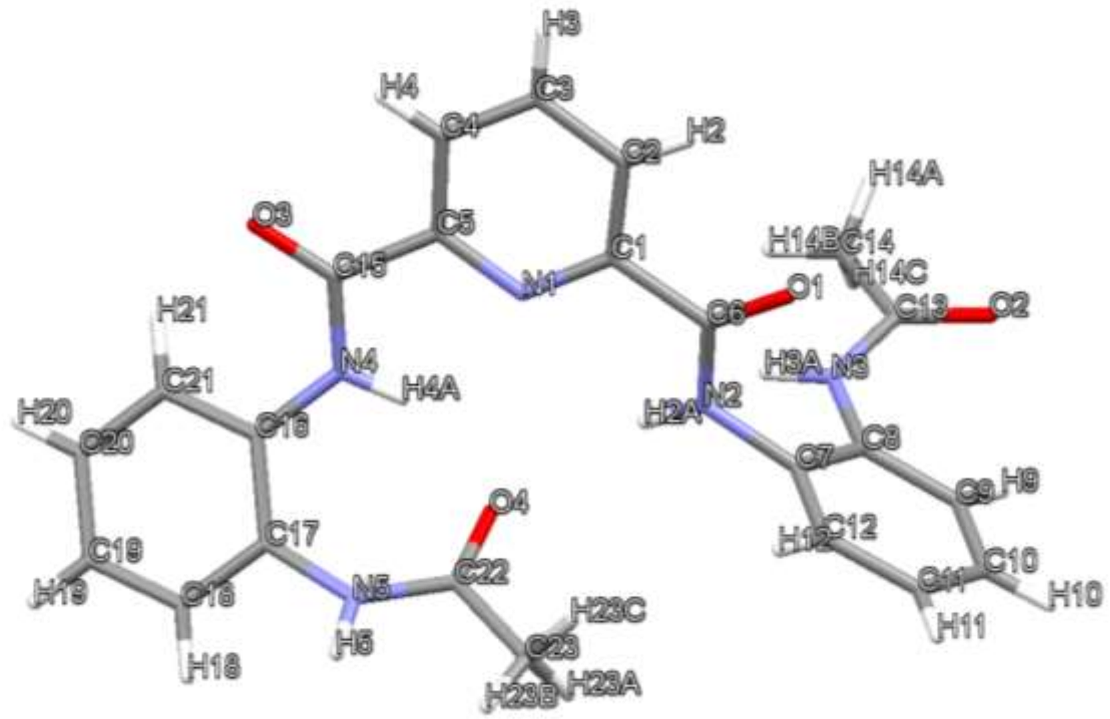

Table 7 Bond Lengths for **1**.

**Atom**	**Atom**	**Length/Å**	**Atom**	**Atom**	**Length/Å**
C1	C2	1.385(2)	C11	C12	1.389(2)
C1	C6	1.503(2)	C13	C14	1.504(2)
C1	N1	1.3329(18)	C13	N3	1.3549(18)
C2	C3	1.381(2)	C13	O2	1.2337(18)
C3	C4	1.387(2)	C15	N4	1.3459(19)
C4	C5	1.389(2)	C15	O3	1.2282(18)
C5	C15	1.5135(19)	C16	C17	1.398(2)
C5	N1	1.3410(19)	C16	C21	1.395(2)
C6	N2	1.3396(19)	C16	N4	1.4105(17)
C6	O1	1.2338(18)	C17	C18	1.3994(19)
C7	C8	1.404(2)	C17	N5	1.4276(19)
C7	C12	1.388(2)	C18	C19	1.383(2)
C7	N2	1.4369(19)	C19	C20	1.385(2)
C8	C9	1.394(2)	C20	C21	1.388(2)
C8	N3	1.4144(19)	C22	C23	1.506(2)
C9	C10	1.388(2)	C22	N5	1.3476(19)
C10	C11	1.386(2)	C22	O4	1.2401(18)

Table 8 Bond Angles for **1**.

Atom	Atom	Atom	Angle/°	Atom	Atom	Atom	Angle/°
C2	C1	C6	117.15(13)	O2	C13	C14	121.59(12)
N1	C1	C2	123.42(14)	O2	C13	N3	123.19(13)
N1	C1	C6	119.44(12)	N4	C15	C5	112.98(12)
C3	C2	C1	119.24(14)	O3	C15	C5	120.15(13)
C2	C3	C4	118.31(14)	O3	C15	N4	126.86(13)
C3	C4	C5	118.37(14)	C17	C16	N4	119.88(13)
C4	C5	C15	118.71(13)	C21	C16	C17	119.33(13)
N1	C5	C4	123.78(13)	C21	C16	N4	120.78(13)
N1	C5	C15	117.49(12)	C16	C17	C18	118.97(14)
N2	C6	C1	117.85(12)	C16	C17	N5	124.74(12)
O1	C6	C1	118.68(13)	C18	C17	N5	116.04(13)
O1	C6	N2	123.43(14)	C19	C18	C17	121.17(14)
C8	C7	N2	119.99(13)	C18	C19	C20	119.79(13)
C12	C7	C8	120.34(13)	C19	C20	C21	119.74(14)
C12	C7	N2	119.35(13)	C20	C21	C16	120.98(14)
C7	C8	N3	118.89(13)	N5	C22	C23	115.11(13)
C9	C8	C7	118.92(13)	O4	C22	C23	120.85(14)
C9	C8	N3	122.18(13)	O4	C22	N5	124.04(14)
C10	C9	C8	120.24(14)	C1	N1	C5	116.83(12)
C11	C10	C9	120.71(14)	C6	N2	C7	118.05(12)
C10	C11	C12	119.50(14)	C13	N3	C8	126.30(12)
C7	C12	C11	120.28(13)	C15	N4	C16	126.50(13)
N3	C13	C14	115.22(12)	C22	N5	C17	129.36(12)

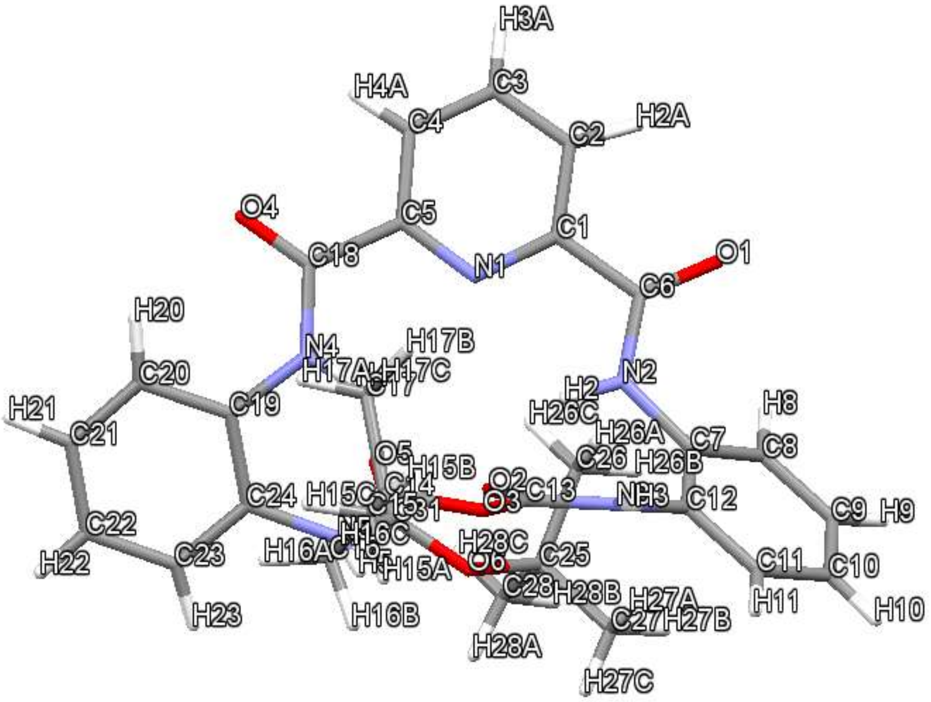

Table 9. Bond Lengths for **2**

**Atom**	**Atom**	**Length/Å**	**Atom**	**Atom**	**Length/Å**
O6	C25	1.468(5)	N2	C7	1.423(5)
O6	C31	1.343(5)	N2	C6	1.336(5)
O5	C31	1.209(5)	C1	C2	1.393(5)
O4	C18	1.228(4)	C1	C6	1.506(5)
O2	C13	1.217(4)	C14	C16	1.517(6)
N5	C24	1.420(5)	C14	C17	1.522(6)
N5	C31	1.356(5)	C14	C15	1.514(6)
O1	C6	1.230(5)	C11	C12	1.393(5)
N3	C13	1.353(5)	C7	C12	1.390(5)
N3	C12	1.416(5)	C7	C8	1.386(6)
O3	C13	1.341(5)	C19	C24	1.401(5)
O3	C14	1.479(5)	C19	C20	1.400(5)
N1	C5	1.342(5)	C2	C3	1.370(6)
N1	C1	1.338(5)	C24	C23	1.388(6)
C10	C11	1.381(6)	C27	C25	1.513(6)
C10	C9	1.376(7)	C22	C23	1.381(6)
N4	C18	1.349(5)	C22	C21	1.370(7)
N4	C19	1.419(5)	C25	C26	1.517(6)
C4	C5	1.374(6)	C25	C28	1.505(6)
C4	C3	1.372(6)	C20	C21	1.374(6)
C18	C5	1.500(5)	C9	C8	1.382(6)

Table 10. Bond Angles for **2**

Atom	Atom	Atom	Angle/°	Atom	Atom	Atom	Angle/°
C31	O6	C25	120.8(3)	C8	C7	C12	119.6(4)
C31	N5	C24	120.2(3)	C24	C19	N4	117.6(3)
C13	N3	C12	127.3(3)	C20	C19	N4	123.6(4)
C13	O3	C14	121.4(3)	C20	C19	C24	118.8(4)
C1	N1	C5	116.8(3)	C11	C12	N3	117.4(4)
C9	C10	C11	119.7(4)	C7	C12	N3	123.8(3)
C18	N4	C19	126.7(3)	C7	C12	C11	118.8(4)
C3	C4	C5	119.2(4)	C3	C2	C1	117.9(4)
O4	C18	N4	124.0(4)	C19	C24	N5	120.1(4)
O4	C18	C5	120.0(4)	C23	C24	N5	119.8(4)
N4	C18	C5	115.9(3)	C23	C24	C19	120.1(4)
N1	C5	C4	123.0(4)	C21	C22	C23	119.2(4)
N1	C5	C18	118.9(3)	O6	C25	C27	101.6(3)
C4	C5	C18	118.0(4)	O6	C25	C26	110.4(4)
C6	N2	C7	124.0(3)	O6	C25	C28	109.6(3)
O2	C13	N3	125.8(4)	C27	C25	C26	111.3(4)
O2	C13	O3	125.5(4)	C28	C25	C27	111.2(4)
O3	C13	N3	108.8(3)	C28	C25	C26	112.2(4)
N1	C1	C2	123.6(4)	C2	C3	C4	119.4(4)
N1	C1	C6	118.3(3)	C21	C20	C19	119.5(4)
C2	C1	C6	118.1(4)	C22	C23	C24	120.4(4)
O3	C14	C16	111.2(4)	O6	C31	N5	108.9(3)
O3	C14	C17	108.7(4)	O5	C31	O6	126.8(4)
O3	C14	C15	102.5(3)	O5	C31	N5	124.4(4)
C16	C14	C17	112.3(4)	C10	C9	C8	119.8(4)
C15	C14	C16	110.9(4)	O1	C6	N2	125.0(4)
C15	C14	C17	110.7(4)	O1	C6	C1	119.9(4)
C10	C11	C12	121.2(4)	N2	C6	C1	115.2(3)
C12	C7	N2	120.2(4)	C22	C21	C20	122.0(4)
C8	C7	N2	120.2(4)	C9	C8	C7	120.9(4)

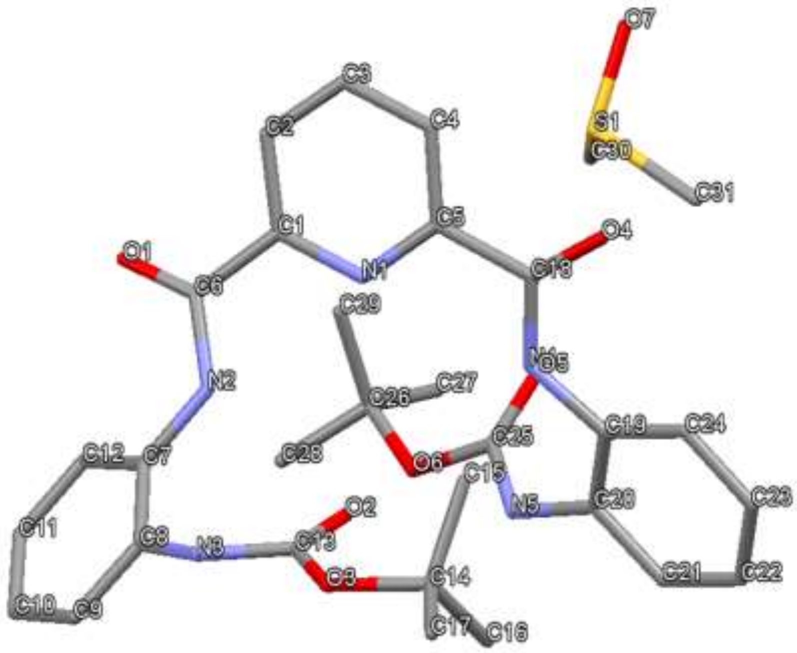

Table 11. Bond Lengths for **2-DMSO**

Atom	Atom	Length/Å	Atom	Atom	Length/Å
S1	O7	1.5022(12)	C6	C11	1.4031(19)
S1	C30	1.7731(16)	C6	C7	1.3867(19)
S1	C31	1.7870(19)	C13	C12	1.5019(19)
O6	C25	1.3470(15)	C13	C14	1.385(2)
O6	C26	1.4817(17)	C19	C20	1.3973(19)
O5	C25	1.2152(16)	C11	C10	1.3920(19)
O3	C12	1.2278(16)	C17	C18	1.499(2)
O4	C18	1.2228(16)	C17	C16	1.391(2)
O1	C5	1.3430(17)	C7	C8	1.387(2)
O1	C1	1.4781(16)	C16	C15	1.382(2)
O2	C5	1.2084(17)	C20	C21	1.382(2)
N5	C25	1.3565(17)	C10	C9	1.388(2)
N5	C24	1.4243(17)	C23	C22	1.382(2)
N1	C6	1.4183(18)	C14	C15	1.383(2)
N1	C5	1.3612(17)	C1	C3	1.516(2)
N3	C13	1.3337(18)	C1	C2	1.517(2)
N3	C17	1.3357(17)	C1	C4	1.513(2)
N2	C11	1.4062(17)	C8	C9	1.377(2)
N2	C12	1.3434(18)	C26	C28	1.510(2)
N4	C19	1.4081(18)	C26	C29	1.516(2)
N4	C18	1.3535(17)	C26	C27	1.517(2)
C24	C19	1.4012(19)	C21	C22	1.379(2)
C24	C23	1.391(2)			

Table 12. Bond Angles for **2-DMSO**

**Atom**	**Atom**	**Atom**	**Angle/°**	**Atom**	**Atom**	**Atom**	**Angle/°**
O7	S1	C30	106.33(8)	N3	C17	C16	122.73(13)
O7	S1	C31	106.08(9)	C16	C17	C18	120.39(12)
C30	S1	C31	97.50(9)	O3	C12	N2	125.02(13)
C25	O6	C26	120.91(11)	O3	C12	C13	121.72(12)
C5	O1	C1	120.57(11)	N2	C12	C13	113.24(12)
C25	N5	C24	125.58(12)	O4	C18	N4	125.42(14)
C5	N1	C6	122.68(13)	O4	C18	C17	121.44(12)
C13	N3	C17	118.22(12)	N4	C18	C17	113.13(11)
C12	N2	C11	128.92(12)	C8	C7	C6	120.79(14)
C18	N4	C19	126.70(12)	C15	C16	C17	118.19(14)
O6	C25	N5	109.20(11)	C21	C20	C19	120.76(14)
O5	C25	O6	125.50(12)	C9	C10	C11	119.89(15)
O5	C25	N5	125.30(12)	C22	C23	C24	120.96(15)
C19	C24	N5	123.47(13)	C15	C14	C13	118.22(15)
C23	C24	N5	117.05(13)	O1	C1	C3	110.39(12)
C23	C24	C19	119.43(12)	O1	C1	C2	109.81(12)
C11	C6	N1	120.88(12)	O1	C1	C4	102.71(12)
C7	C6	N1	119.71(13)	C3	C1	C2	112.63(14)
C7	C6	C11	119.28(12)	C4	C1	C3	110.64(14)
N3	C13	C12	116.48(12)	C4	C1	C2	110.21(14)
N3	C13	C14	123.06(13)	C9	C8	C7	119.70(14)
C14	C13	C12	120.42(13)	C8	C9	C10	120.62(14)
O1	C5	N1	109.04(12)	O6	C26	C28	109.57(12)
O2	C5	O1	126.44(13)	O6	C26	C29	110.20(12)
O2	C5	N1	124.52(13)	O6	C26	C27	102.33(12)
C24	C19	N4	120.03(12)	C28	C26	C29	112.84(15)
C20	C19	N4	121.10(13)	C28	C26	C27	110.39(14)
C20	C19	C24	118.86(13)	C29	C26	C27	111.00(15)
C6	C11	N2	117.37(12)	C22	C21	C20	120.22(15)
C10	C11	N2	122.81(13)	C21	C22	C23	119.73(16)
C10	C11	C6	119.69(13)	C16	C15	C14	119.53(14)
N3	C17	C18	116.86(12)				

Table 13 Bond Lengths for **3.**

Atom	Atom	Length/Å	Atom	Atom	Length/Å
N6	C24	1.325(5)	N2	C7	1.428(5)
N6	C20	1.347(5)	N2	C6	1.342(5)
N9	C33	1.423(5)	N1	C1	1.330(5)
N9	C32	1.347(5)	N1	C5	1.358(5)
N7	C25	1.360(5)	O2	C13	1.243(5)
N7	C26	1.400(5)	C1	C6	1.517(6)
C33	C38	1.404(5)	C1	C2	1.394(6)
C33	C34	1.357(6)	N3	C12	1.379(6)
C38	C37	1.382(6)	O1	C6	1.219(5)
C38	N10	1.385(6)	N4	C14	1.392(5)
C32	C24	1.507(6)	N4	C13	1.355(5)
C32	O4	1.217(4)	C5	C4	1.398(6)
C37	C36	1.373(7)	C5	C13	1.471(6)
C24	C23	1.401(6)	C12	C7	1.396(6)
C20	C25	1.479(6)	C12	C11	1.409(6)
C20	C21	1.405(6)	C7	C8	1.369(6)
O3	C25	1.225(5)	C4	C3	1.369(7)
C34	C35	1.393(7)	C11	C10	1.362(7)
C21	C22	1.365(7)	C8	C9	1.394(7)
C22	C23	1.382(7)	C14	C19	1.408(6)
C26	C31	1.415(6)	C14	C15	1.379(6)
C26	C27	1.387(6)	C2	C3	1.375(6)
C36	C35	1.375(7)	N5	C19	1.385(6)
N8	C31	1.397(5)	C19	C18	1.371(6)
C31	C30	1.371(6)	C15	C16	1.390(8)
C27	C28	1.395(7)	C9	C10	1.375(8)
C28	C29	1.400(8)	C18	C17	1.343(8)
C30	C29	1.360(7)	C16	C17	1.398(10)

Table 14 Bond Angles for **3.**

**Atom**	**Atom**	**Atom**	**Angle/°**	**Atom**	**Atom**	**Atom**	**Angle/°**
C24	N6	C20	117.8(3)	C6	N2	C7	128.6(4)
C32	N9	C33	128.4(4)	C1	N1	C5	117.7(3)
C25	N7	C26	128.5(4)	N1	C1	C6	117.1(3)
C38	C33	N9	117.3(4)	N1	C1	C2	122.7(4)
C34	C33	N9	122.5(4)	C2	C1	C6	120.2(4)
C34	C33	C38	120.0(4)	C13	N4	C14	127.3(4)
C37	C38	C33	118.1(4)	N1	C5	C4	122.6(4)
C37	C38	N10	121.8(4)	N1	C5	C13	116.6(3)
N10	C38	C33	119.9(4)	C4	C5	C13	120.8(4)
N9	C32	C24	113.7(4)	N3	C12	C7	121.4(4)
O4	C32	N9	125.3(4)	N3	C12	C11	120.3(4)
O4	C32	C24	121.0(4)	C7	C12	C11	118.2(4)
C36	C37	C38	122.0(5)	C12	C7	N2	117.3(4)
N6	C24	C32	117.3(3)	C8	C7	N2	121.9(4)
N6	C24	C23	123.0(4)	C8	C7	C12	120.8(4)
C23	C24	C32	119.7(4)	C3	C4	C5	118.1(4)
N6	C20	C25	117.2(3)	N2	C6	C1	114.2(4)
N6	C20	C21	122.7(4)	O1	C6	N2	126.1(4)
C21	C20	C25	120.2(4)	O1	C6	C1	119.6(4)
C33	C34	C35	120.9(5)	C10	C11	C12	121.1(5)
N7	C25	C20	114.5(4)	C7	C8	C9	119.5(5)
O3	C25	N7	124.2(4)	N4	C14	C19	117.3(4)
O3	C25	C20	121.2(4)	C15	C14	N4	122.8(4)
C22	C21	C20	118.4(4)	C15	C14	C19	119.9(4)
C21	C22	C23	119.7(4)	O2	C13	N4	124.2(4)
C22	C23	C24	118.4(4)	O2	C13	C5	120.8(4)
N7	C26	C31	116.9(4)	N4	C13	C5	115.0(4)
C27	C26	N7	122.2(4)	C3	C2	C1	118.7(5)
C27	C26	C31	120.9(4)	C4	C3	C2	120.1(4)
C37	C36	C35	119.1(5)	N5	C19	C14	119.9(4)
N8	C31	C26	120.4(4)	C18	C19	C14	118.9(5)
C30	C31	C26	117.8(4)	C18	C19	N5	121.2(5)
C30	C31	N8	121.8(4)	C14	C15	C16	120.4(6)
C36	C35	C34	119.8(5)	C10	C9	C8	120.8(5)
C26	C27	C28	119.5(5)	C11	C10	C9	119.6(5)
C27	C28	C29	119.1(5)	C17	C18	C19	121.2(6)
C29	C30	C31	122.3(5)	C15	C16	C17	118.2(6)
C30	C29	C28	120.4(5)	C18	C17	C16	121.4(6)

Solid State Analysis of **1**

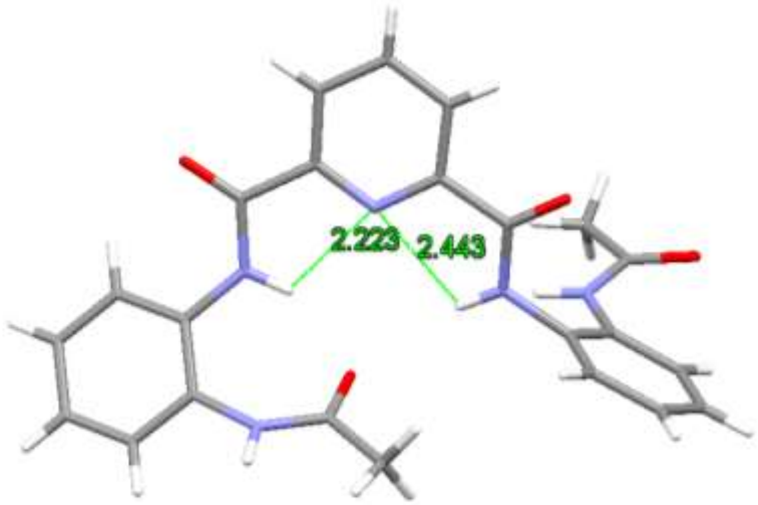

Figure S4. Solid state analysis of **1** highlighting the presence of bifurcated intramolecular N-H···N hydrogen bonding interactions involving the N atom of the pyridine and the two NH's of the adjacent amide bonds.[10]

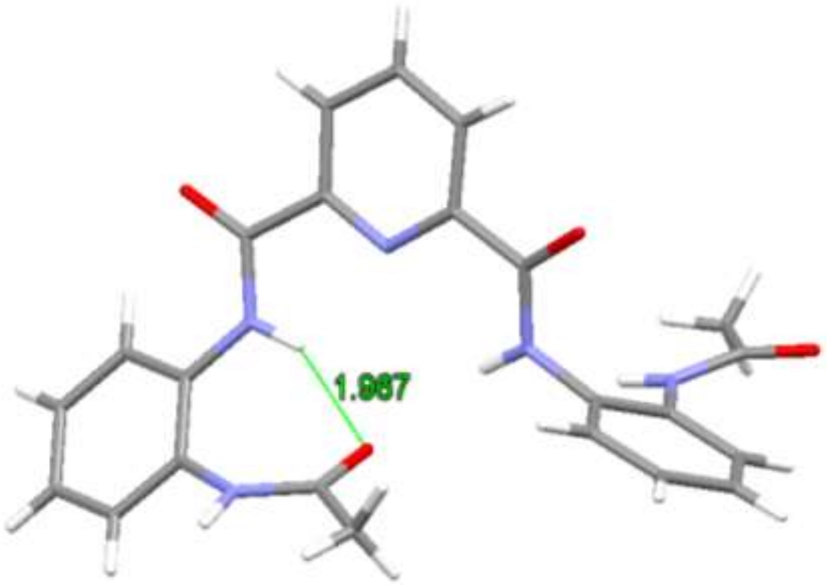

Figure S5. Solid state analysis of **1** highlighting the presence of an intramolecular N-H···O=C hydrogen bonding interaction involving an NH of one of the central amide bonds and the O atom of the carbonyl group on one of the terminal Ac groups.

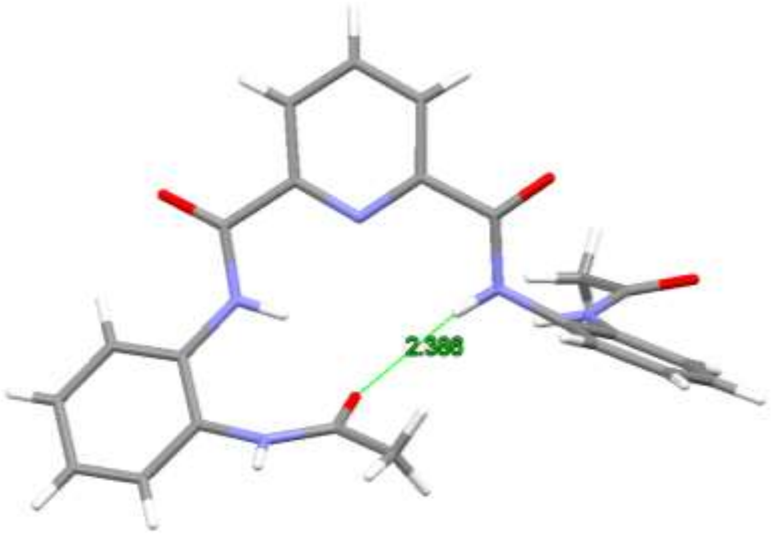

Figure S6. Solid state analysis of **1** highlighting the presence of an intramolecular N-H···O=C hydrogen bonding interaction involving an NH of one of the central amide bonds and the O atom of the carbonyl group on one of the terminal Ac groups.

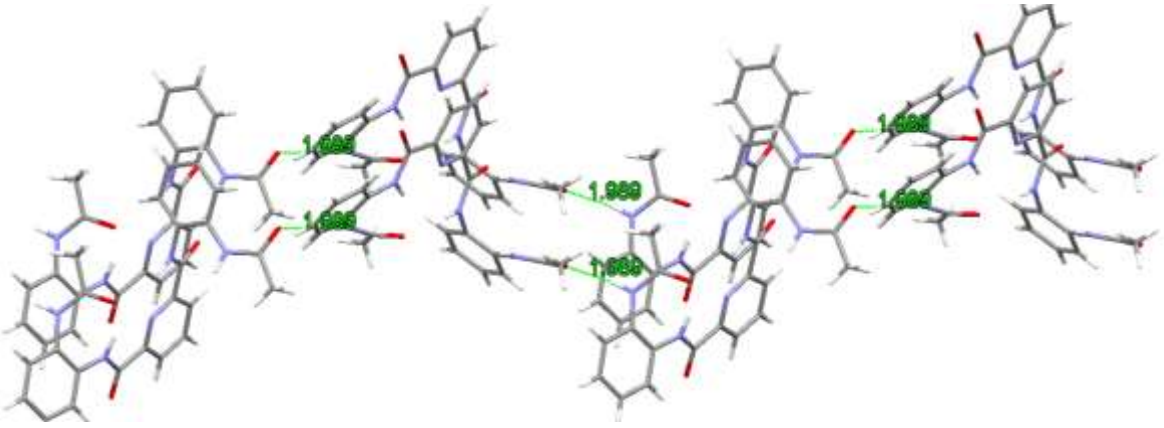

Figure S7. Solid state analysis of **1** highlighting the presence of intermolecular N-H···O=C hydrogen bonding interactions aligned along the *c* axis involving the NHs in the terminal Ac group and the O atom of the carbonyl group in the terminal Ac group in an adjacent molecule.

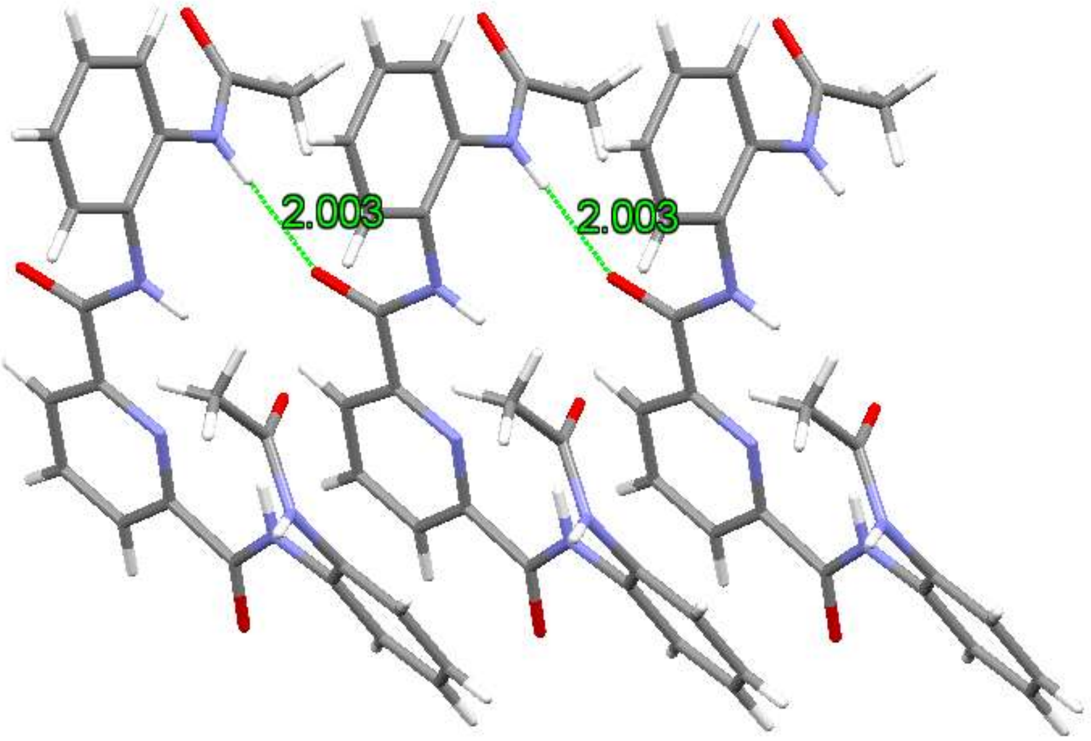

Figure S8. Solid state analysis of **1** highlighting the presence of an intermolecular N-H···O=C hydrogen bonding interaction involving an NH in one of the terminal Ac groups and the O atom of the carbonyl group in the central amide bond.

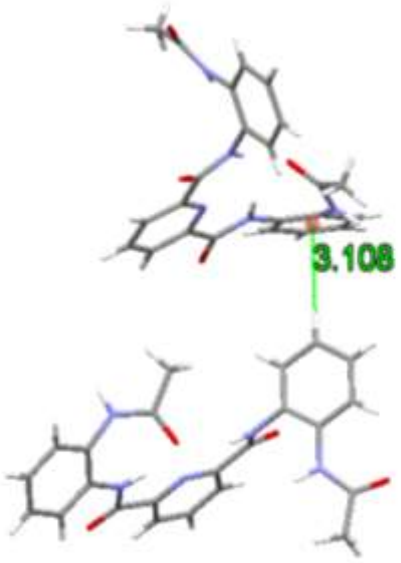

Figure S9. Solid state analysis of **1** highlighting the presence of an intermolecular edge-to-face π-π stacking interaction between the terminal 2-acetylcarboxyaminophenyl rings on adjacent molecules.

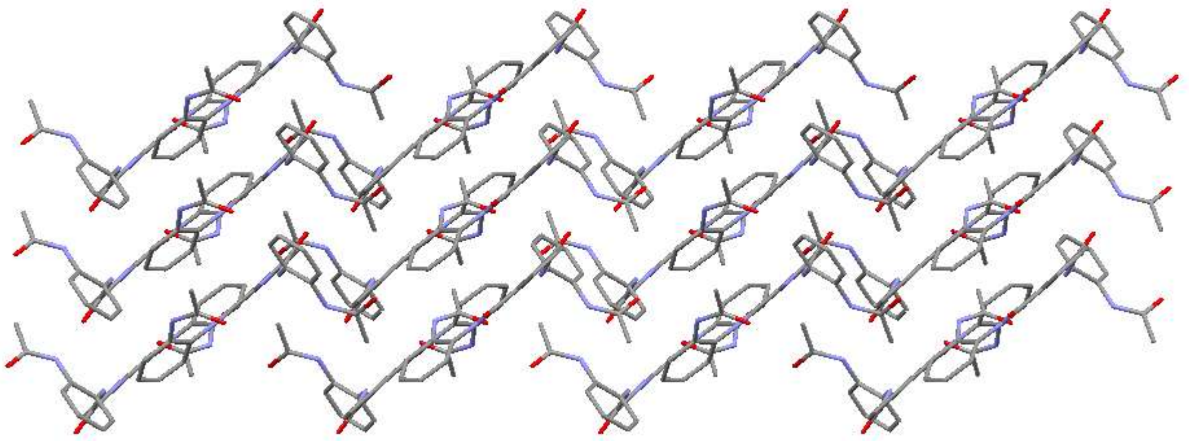

Figure S10. Solid state analysis of **1** highlighting the slipped stack crystal packing arrangement.[11]

Solid State Analysis of **2**

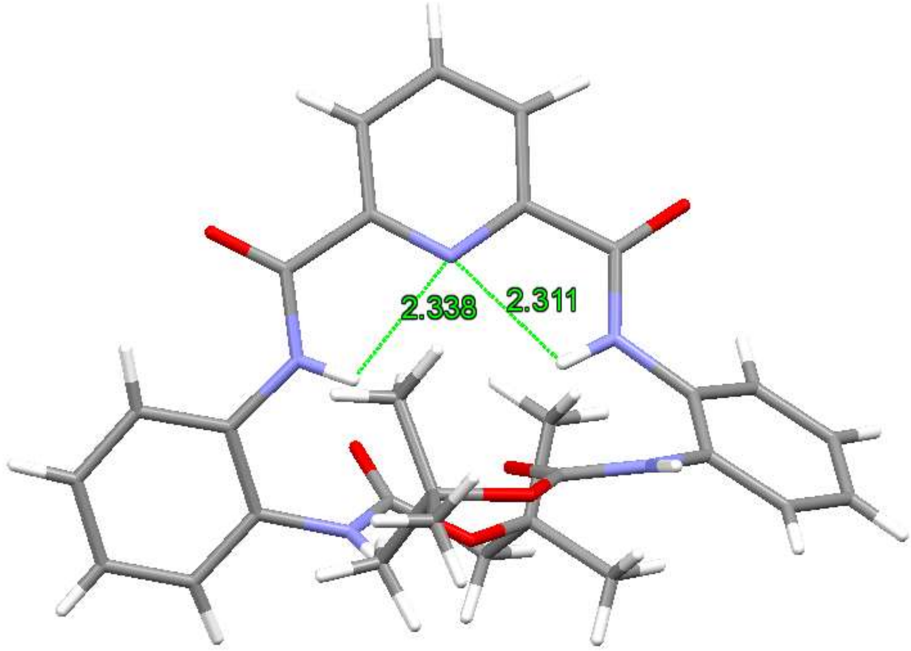

Figure S11. Solid state analysis of **2** highlighting the presence of bifurcated intramolecular N-H···N hydrogen bonding interactions[10] involving the N atom of the pyridine and the two NH's of the adjacent amide bonds.

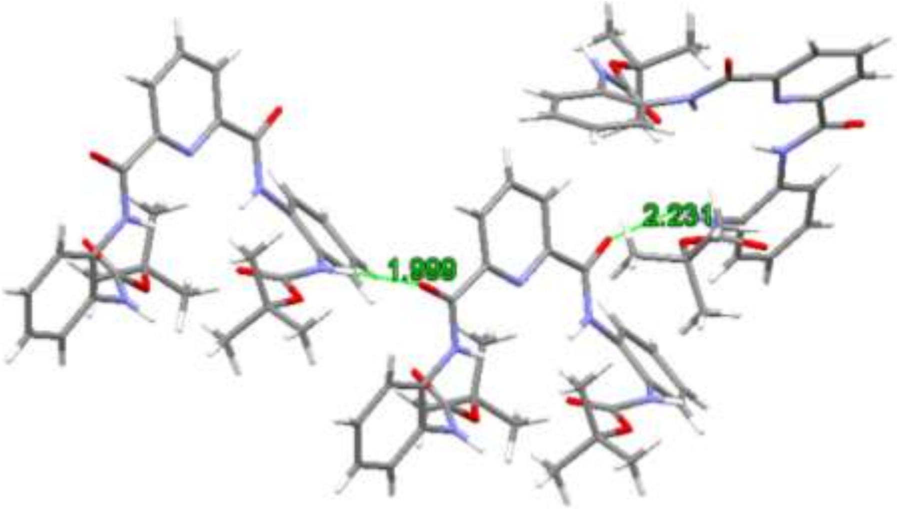

Figure S12. Solid state analysis of **2** highlighting the presence of intermolecular N-H···O=C hydrogen bonding interactions involving the NHs in the terminal Boc group and the O atom of the carbonyl group next to the pyridine ring.

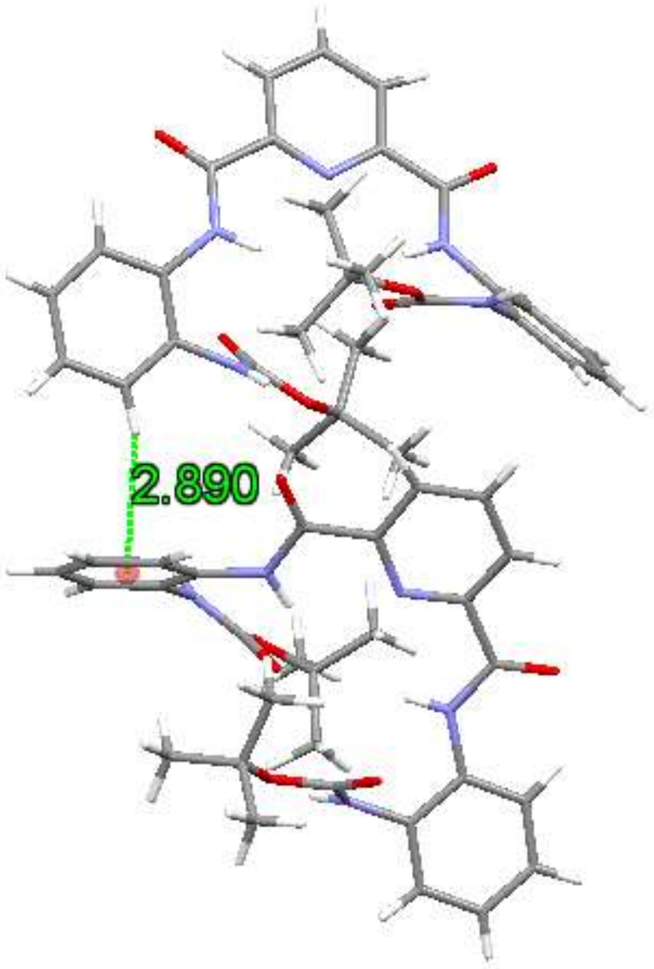

Figure S13. Solid state analysis of **2** highlighting the presence of intermolecular edge-to-face π-π stacking interactions.[12]

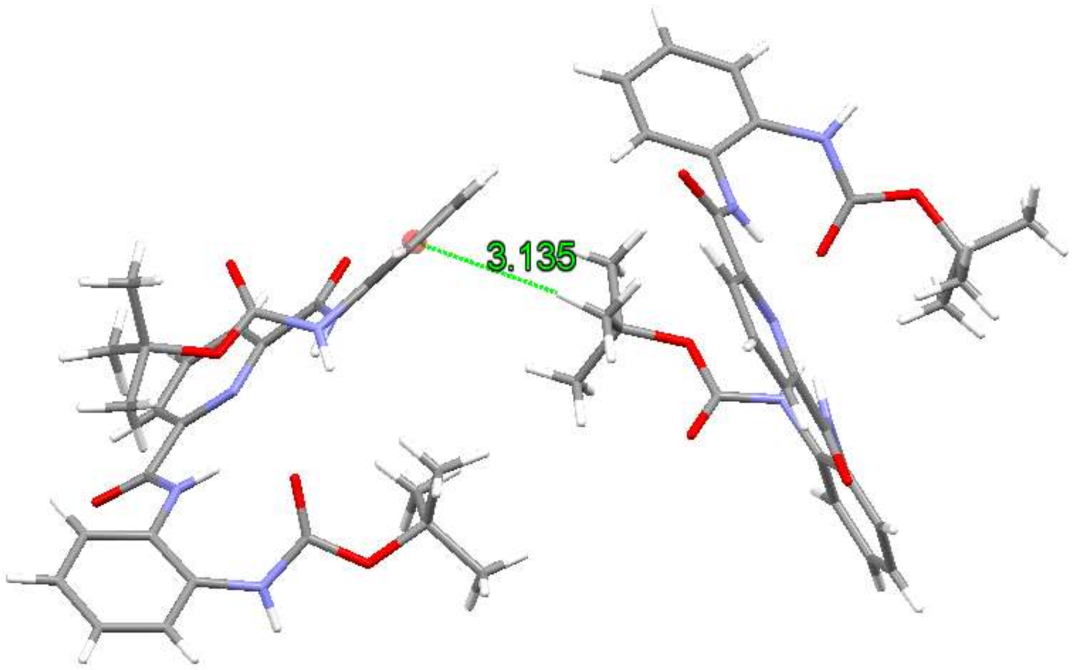

Figure S14. Solid state analysis of **2** highlighting the presence of an intermolecular C-H(aryl)··· π interaction involving an H atom of the Boc group and a terminal 2-*tert*-butylcarboxyaminophenyl ring of an adjacent molecule.[13]

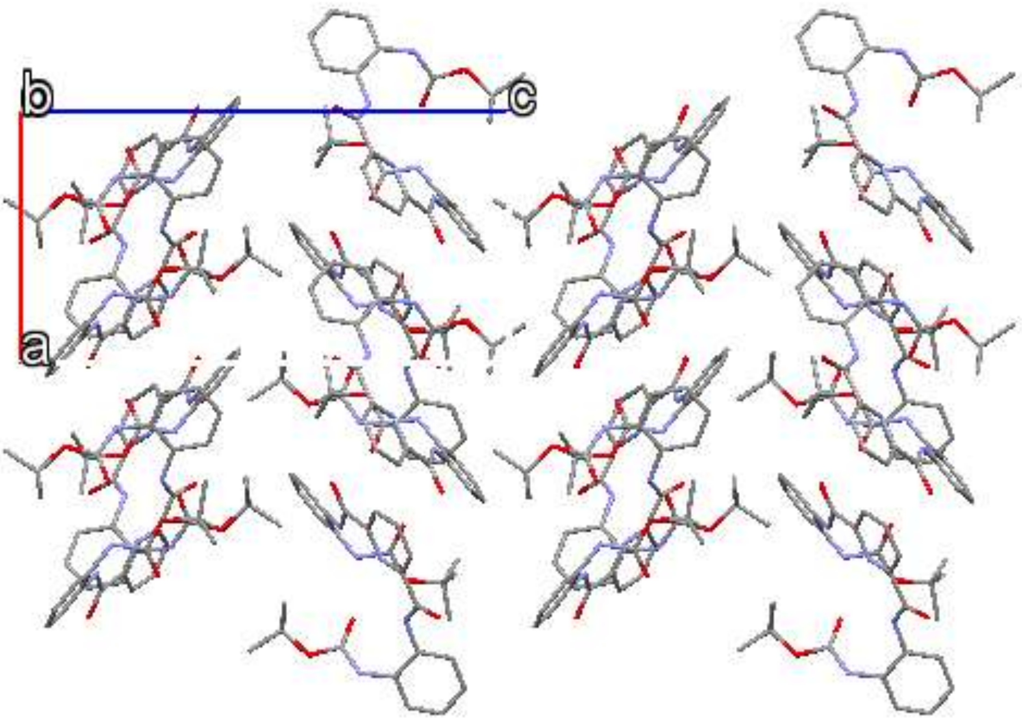

Figure S15. Solid state analysis of **2** highlighting the herringbone crystal packing arrangement.[14]

Solid State Analysis of **2-DMSO**

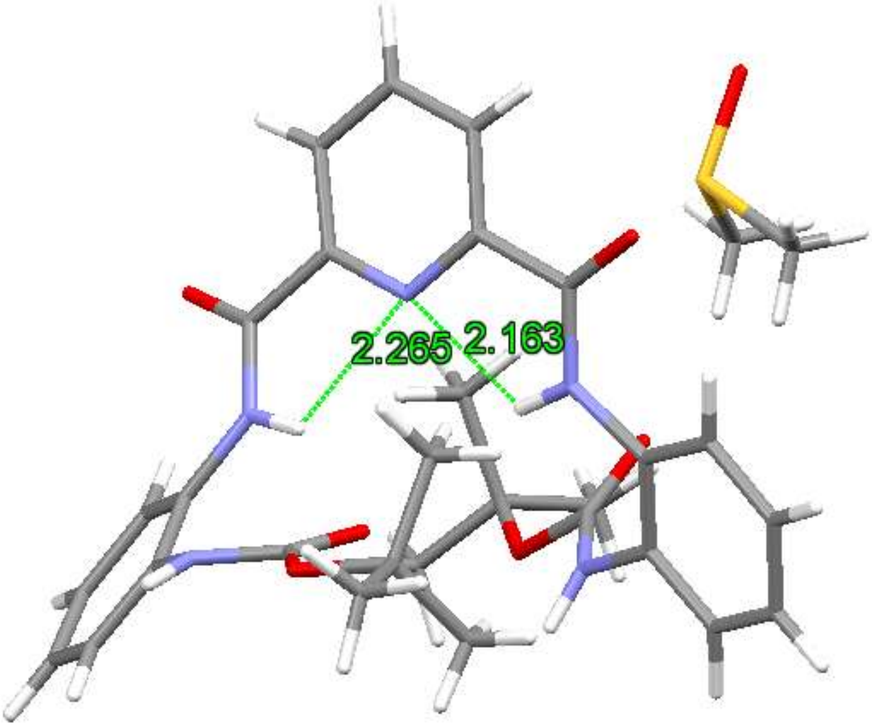

Figure S16. Solid state analysis of **2-**DMSO highlighting the presence of bifurcated intramolecular N-H···N hydrogen bonding interactions[10] involving the N atom of the pyridine and the two NH's of the adjacent amide bonds.

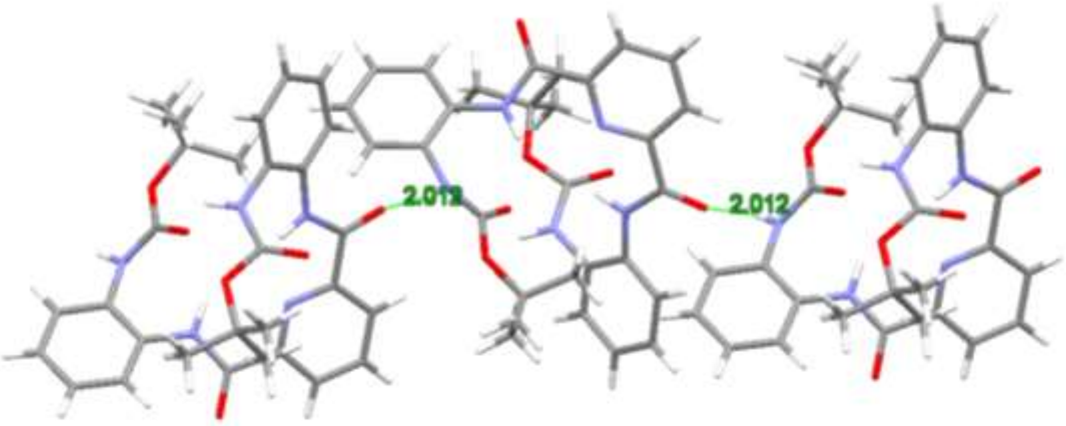

Figure S17. Solid state analysis of **2-**DMSO highlighting the presence of intermolecular N-H···O=C hydrogen bonding interactions involving the NHs in the terminal Boc group and the O atom of the carbonyl group next to the pyridine ring. DMSO molecule removed for clarity.

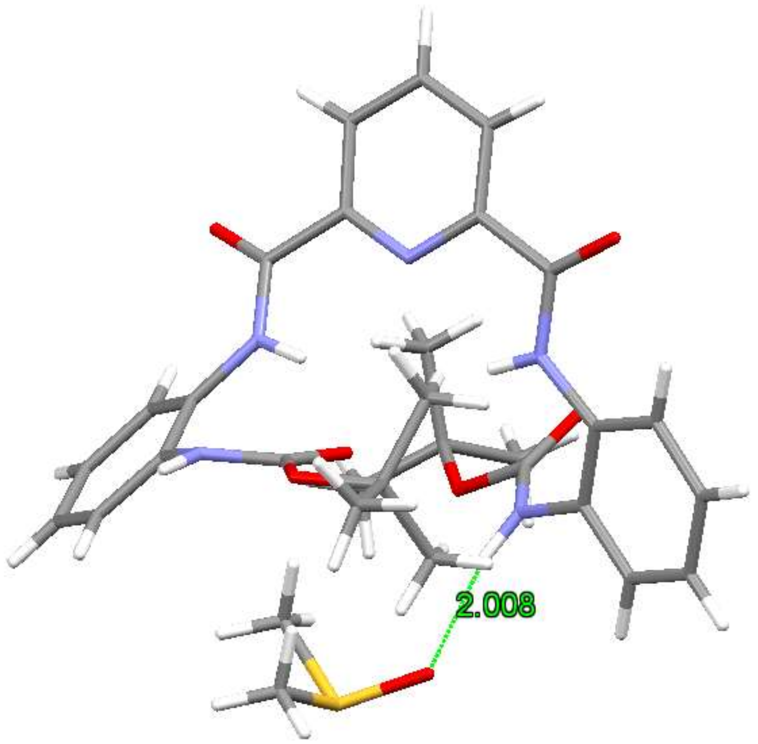

Figure S18. Solid state analysis of **2·**DMSO highlighting the presence of intermolecular N-H···O=C hydrogen bonding interactions involving the NHs in the terminal Boc group and the O atom of the carbonyl group next to the pyridine ring.[15]

Solid State Analysis of **3**

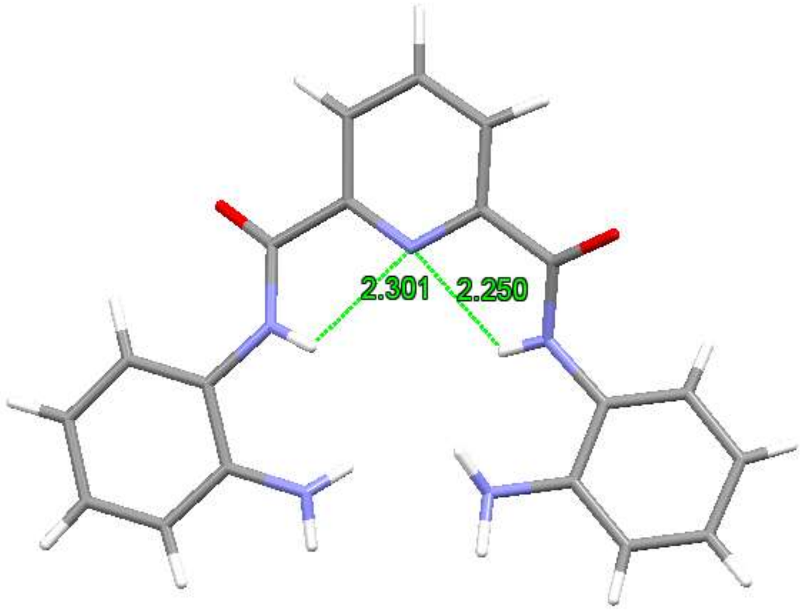

Figure S19. Solid state analysis of one of the molecules in the unit cell of **3** highlighting the presence of bifurcated intramolecular N-H···N hydrogen bonding interactions[10] involving the N atom of the pyridine and the two NH's of the adjacent amide bonds.

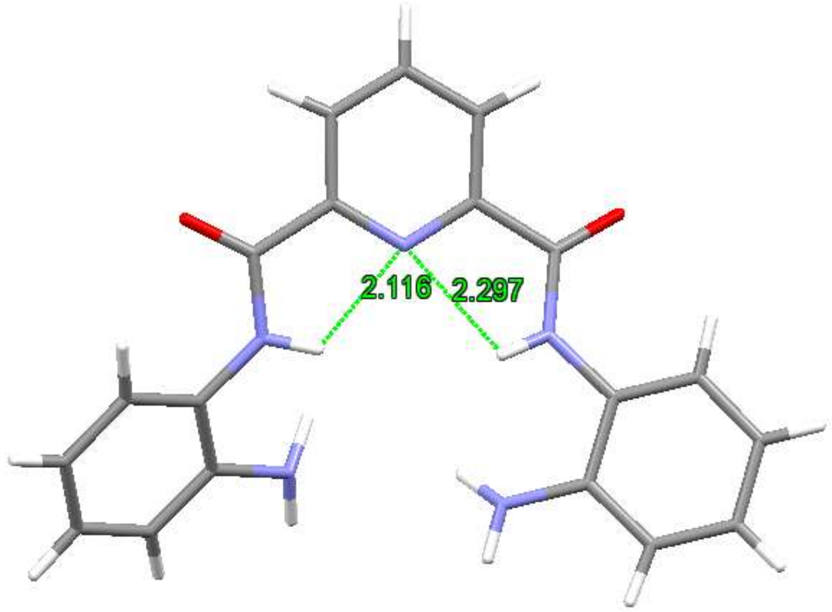

Figure S20. Solid state analysis of one of the molecules in the unit cell of **3** highlighting the presence of bifurcated intramolecular N-H···N hydrogen bonding interactions[10] involving the N atom of the pyridine and the two NH's of the adjacent amide bonds.

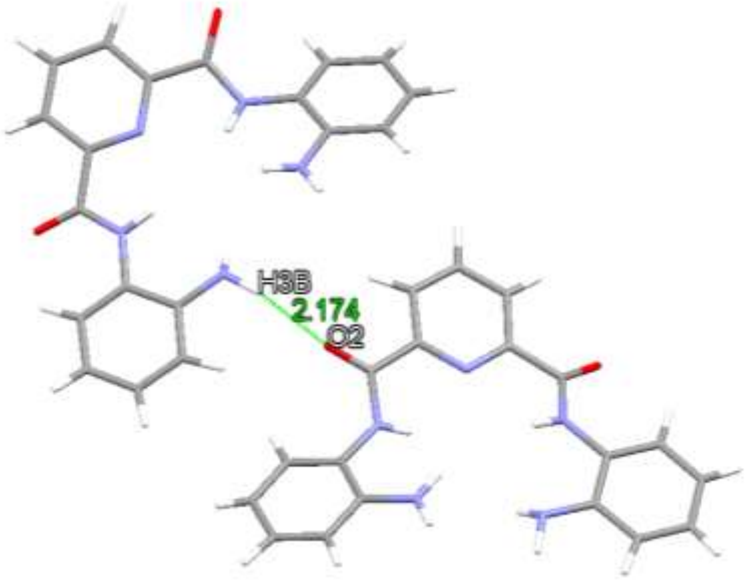

Figure S21. Solid state analysis of **3** highlighting the presence of an intermolecular N-H···O=C hydrogen bonding interaction involving the terminal amine functionality and the O atom of the carbonyl group in the amide bond of adjacent molecule.

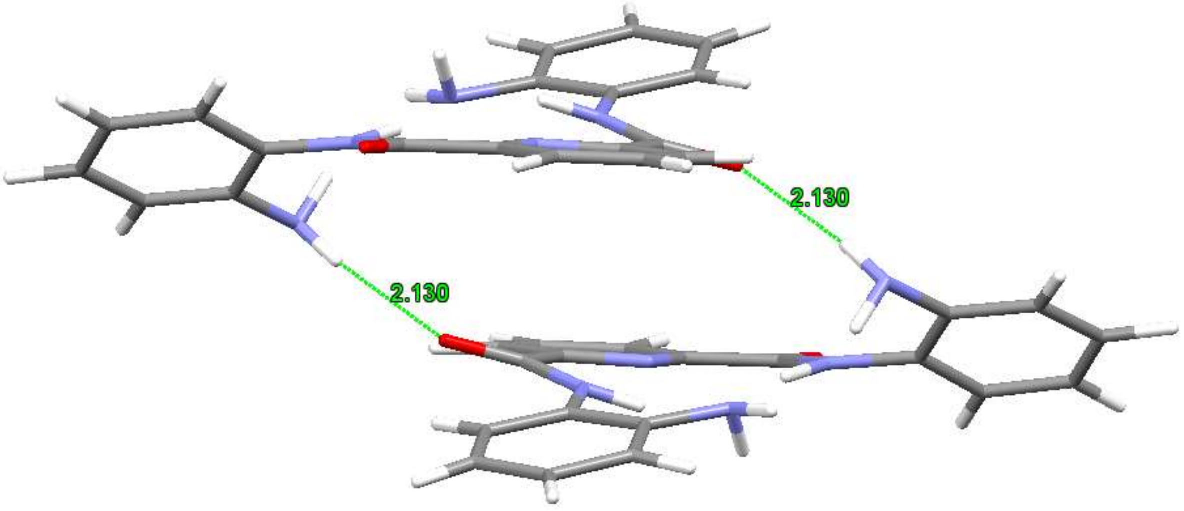

Figure S22. Solid state analysis of **3** highlighting the presence of reciprocal intermolecular N-H···O=C hydrogen bonding interactions involving the terminal amine functionality and the O atom of the carbonyl group in the amide bond of adjacent molecule.

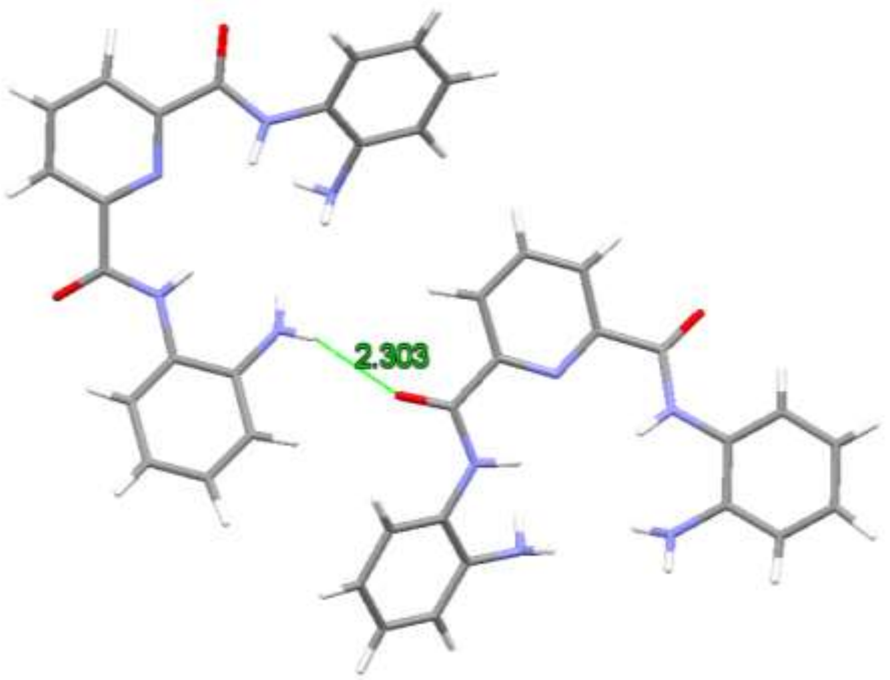

Figure S23. Solid state analysis of **3** highlighting the presence of an intermolecular N-H···O=C hydrogen bonding interaction involving the terminal amine functionality and the O atom of the carbonyl group in the amide bond of adjacent molecule.

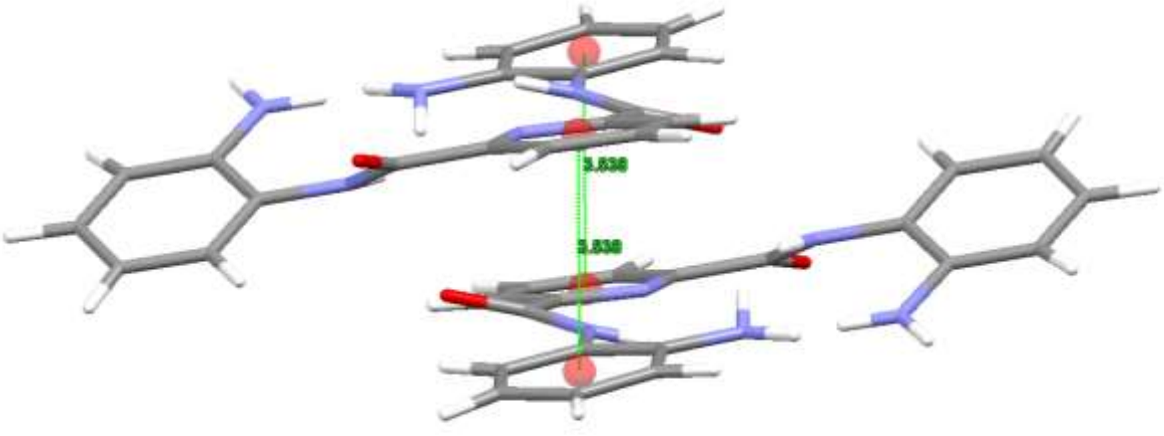

Figure S24. Solid state analysis of **3** highlighting the presence of intermolecular off-set face-to-face π-π stacking interactions between the central pyridine ring and a terminal aminophenyl ring between two adjacent molecules.[16]

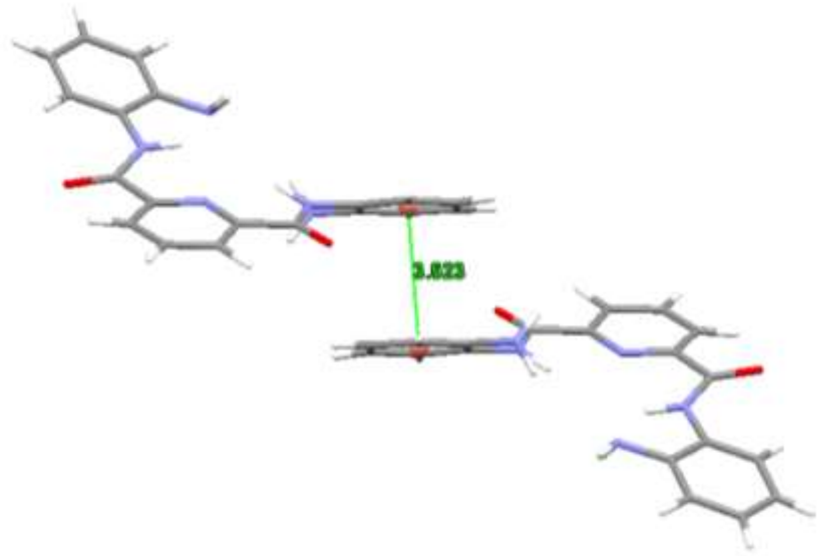

Figure S25. Solid state analysis of **3** highlighting the presence of intermolecular off-set face-to-face π-π stacking interactions between two terminal aminophenyl rings in adjacent molecules.[17]

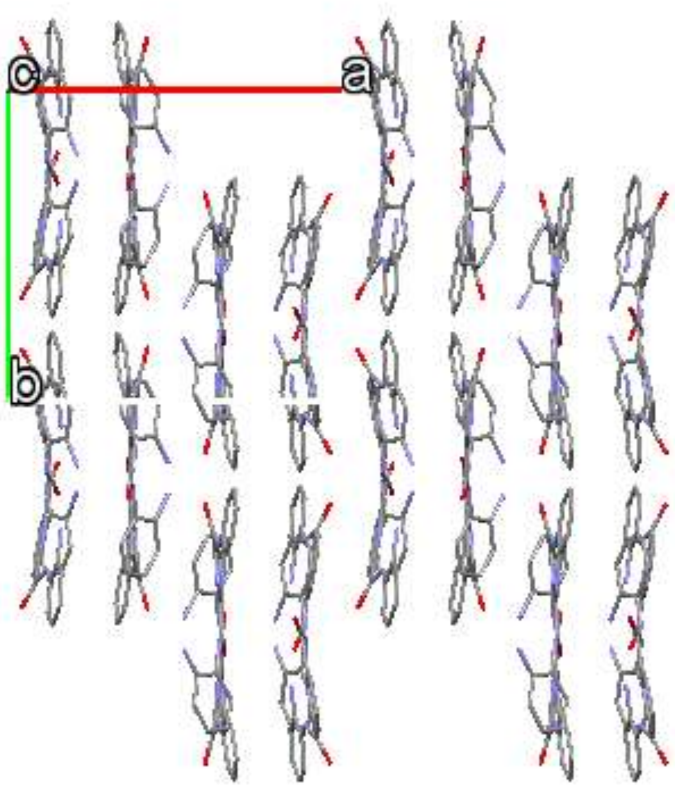

Figure S25. Solid state analysis of **1** highlighting the cofacial and slipped stack crystal packing arrangement as viewed along the *c* axis.[11,17]

Appendix: ^{1}H and ^{13}C NMR spectra

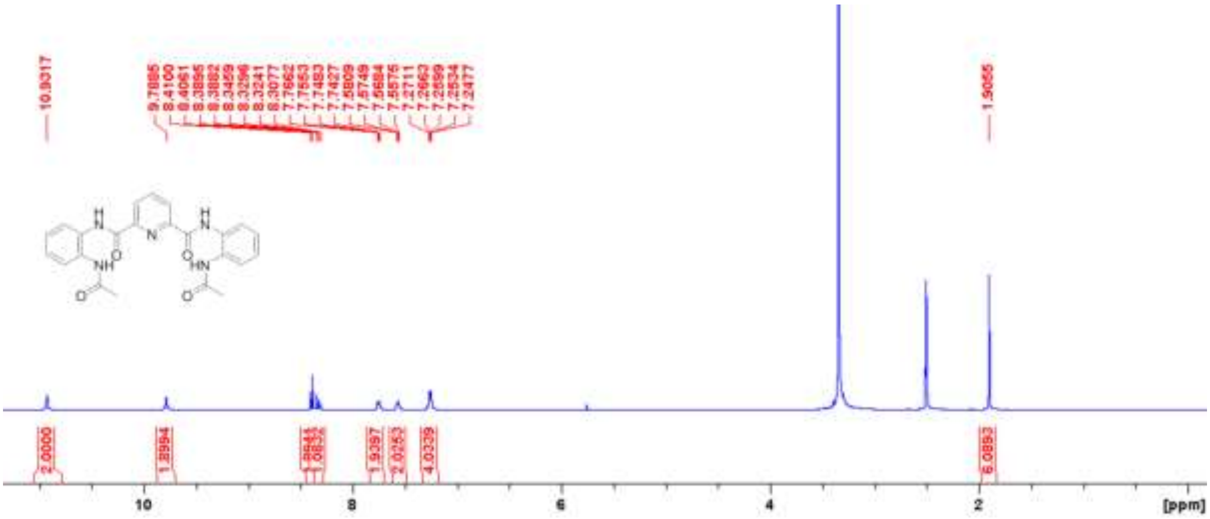

^{1}H NMR spectrum (400 MHz, $(CD_3)_2SO$, 298 K) of 1.

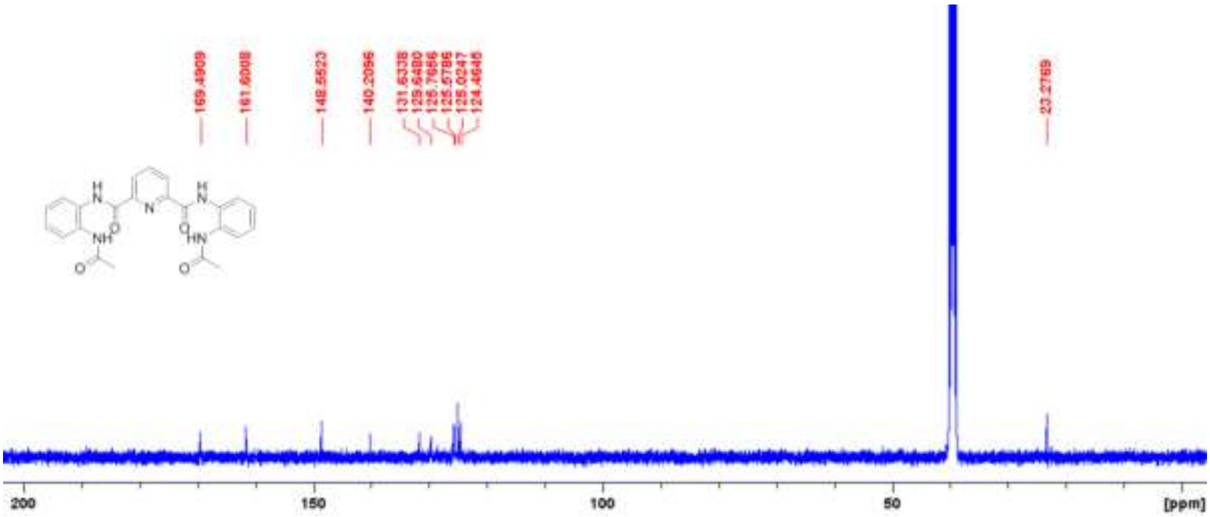

^{13}C NMR spectrum (100 MHz, $(CD_3)_2SO$, 298 K) of 1.

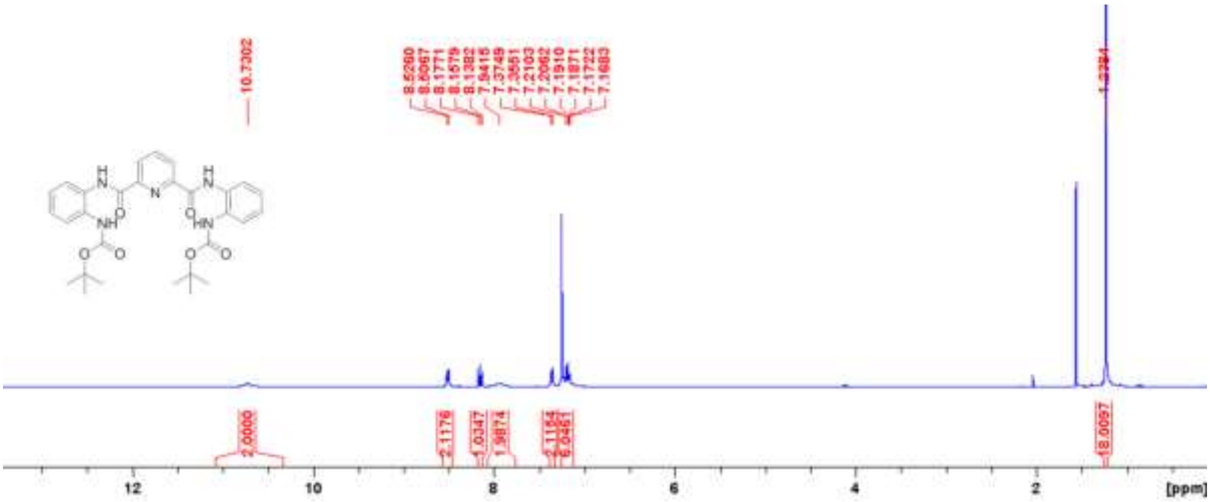

^{1}H NMR spectrum (400 MHz, $CDCl_3$, 298 K) of 2.

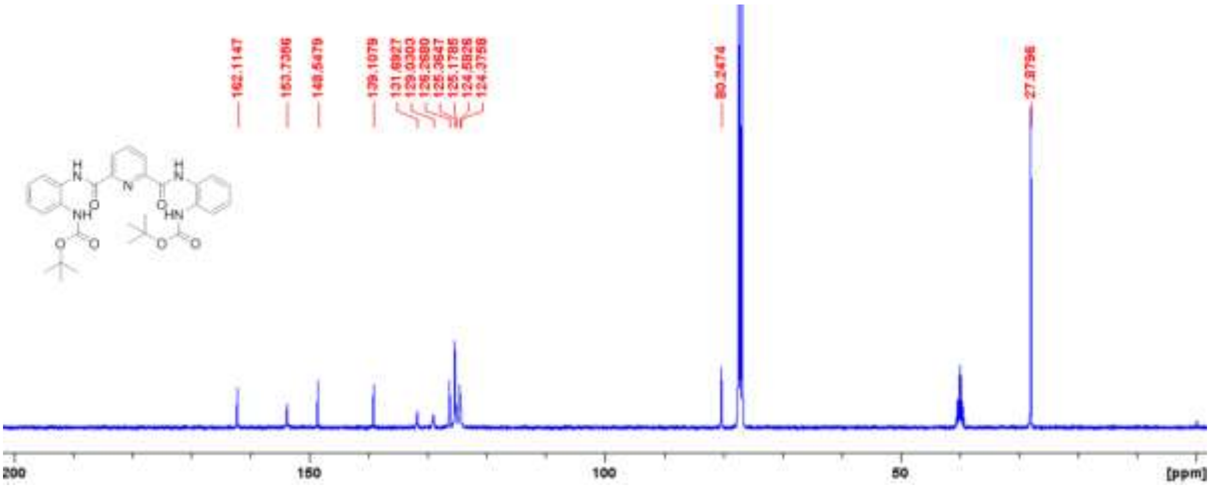

^{13}C NMR spectrum (100 MHz, $CDCl_3$, 298 K) of 2.

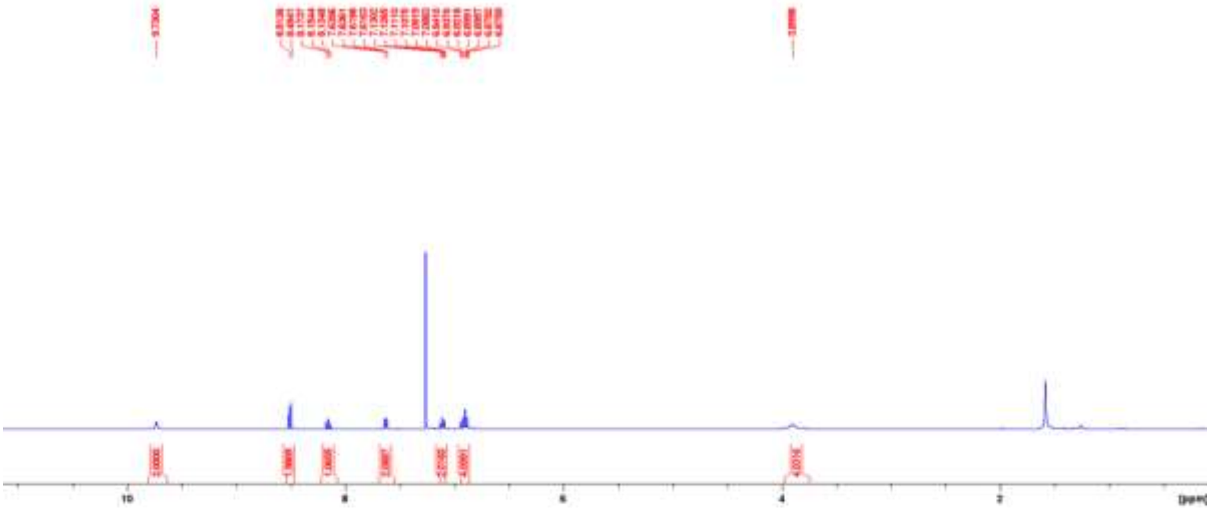

^{1}H NMR spectrum (400 MHz, $CDCl_3$, 298 K) of 3.

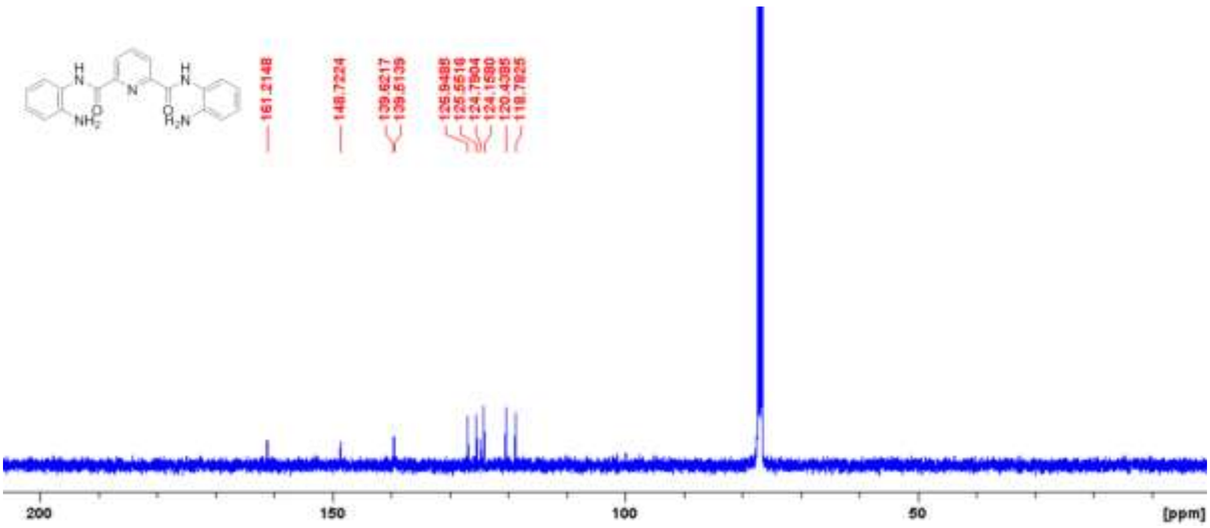

^{13}C NMR spectrum (100 MHz, $CDCl_3$, 298 K) of 3.

References

[1] S. Bellotto, R. Reuter, C. Heinis, H. A. Wegner, *J. Org. Chem.*, **2011**, *23*, 9826.

[2] A. H. Shinde, S. Vidyacharan, D. S. Sharada, *Org. Biomol. Chem.*, **2016**, *14*, 3207.

[3] CrysAlisPro, Rigaku Oxford Diffraction, **2015**.

[4] SAINT v8.34A, Bruker, **2013**.

[5] SADABS-2014/5, Bruker, **2014/5**.

[6] G. M. Sheldrick, *Acta Cryst.*, **2008**, *A64*, 112-122.

[7] G. M. Sheldrick, *Acta Cryst.* **2015**, *A71*, 3-8.

[8] G. M. Sheldrick, *Acta Cryst.* **2015**, *C71*, 3-8.

[9] O. V. Dolomanov, L. J. Bourhis, R. J. Gildea, J. A. K. Howard, H. Puschmann, *J. Appl. Crystallogr.* **2009**, *42*, 339-341.

[10] I. Rozas, I. Alkorta and J. Elguero, *J. Phys. Chem. A.*, **1998**, *102*, 9925.

[11] a) Z.-F. Yao, J.-Y. Wang, J. Pei, *Cryst. Growth Des.*, **2018**, *1*, 7; b) Y.-C. Chang, Y.-D. Chen, C.-H. Chen, Y.-S. Wen, J. T. Lin, H.-Y. Chen, M.-Y. Kuo, I. Chao, *J. Org. Chem.*, **2008**, *12*, 4608.

[12] M. Nishio, *Phys. Chem. Chem. Phys.*, **2011**, *13*, 13873.

[13] G. Tárkányi, P. Király, S. Varga, B. Vakulya, T. Soós, *Chem. Eur. J.*, **2008**, *14*, 6078.

[14] J. Dhar, N. Venkatramaiah, A. Anitha, S. Patil, *J. Mater. Chem. C*, **2014**, *2*, 3457.

[15] M. Arifuzzaman, T. A. Siddique, M. R. Karim, A. H. Mirza, M. A. Ali, *Crystal Structure Theory and Applications*, **2013**, *2*, 159.

[16] M. Egli, V. Tereshko, G. N. Mushudov, R. Sunushvilli, X. Liu, F. D. Lewis, *J. Am. Chem. Soc.*, **2003**, *125*, 10842.

[17] K. Kobayashi, R. Shimaoka, M. Kawahata, M. Yamanaka, K. Yamaguchi, *Org. Lett.* **2006**, *8*, 2385.
